# Sexual dichromatism and color diversity in the spiny lava lizard *Tropidurus spinulosus* using lizard visual modelling

**DOI:** 10.1038/s41598-019-50712-0

**Published:** 2019-10-03

**Authors:** N. Rossi, S. Benitez-Vieyra, A. Cocucci, M. Chiaraviglio, G. Cardozo

**Affiliations:** 1 Universidad Nacional de Córdoba, Facultad de Ciencias Exactas Físicas y Naturales. Laboratorio de Biología del Comportamiento, Córdoba, Argentina; 2 Consejo Nacional de Investigaciones Científicas y Técnicas (CONICET), Instituto de Diversidad y Ecología Animal (IDEA), Córdoba, Argentina; 3 Laboratorio de Ecología Evolutiva y Biología Floral, IMBIV-CONICET, FCEFyN, Universidad Nacional de Córdoba, Córdoba, Argentina

**Keywords:** Sexual selection, Animal behaviour, Herpetology

## Abstract

Colors are important vehicles for social signals in many taxa. In Squamata, previous studies have linked color characteristics and chromatic diversity to sexual selection and, particularly, species showing male-biased body size dimorphism also showed male-biased dichromatism and color diversity. Sexual dichromatism may occur in body regions used for conspecific communication and it may be expressed at wavelengths, such as ultraviolet, easily perceivable by conspecifics. We tested this prediction in a social lizard model, *Tropidurus spinulosus*, using spectrophotometry and visual modelling which enable colors to be interpreted as the individuals of the same taxon see them. Our results indicate that sexual dichromatism occurs in the ventral regions and the flanks, which are the body regions involved in sexual displays. Males show greater color diversity, having larger color volumes and more contrasting colors. These findings reinforce the idea that sexual selection towards males is coupled with the evolution of male-biased, diverse, coloration which could act as a signal in social reproductive contexts.

## Introduction

Colors often act as social signals in many taxa^1^. Color signals evolved to be clearly distinguishable by the visual system of intended receivers and provide information that can be used to take behavioural decisions^2,3^. In lizards they may be used in the recognition of conspecifics, mate choice and intra-sexual interactions^4,5^. Color patterns are subject to both ecological and sexual pressures^6,7^, which may act differentially on sexes, leading to different colorations (hereafter, sexual dichromatism)^8^. In some taxa, such as Lacertidae and Agamidae the degree of dichromatism is positively associated with sexual size dimorphism^7,9,10^, a classical proxy of the intensity of sexual selection in lizards since in many species it has been related to both intrasexual (male-male interactions) and intersexual (mate choice) dynamics^11,12^. From a macroevolutionary perspective, sexual size dimorphism of the whole-body size and of body parts is influenced by sexual selection shaping sexual phenotype diversity^13^. In Agamidae the association also extends to the color diversity and pattern complexity of males^9^ i.e.sexual dichromatism may show distinct and contrasting colors, suggesting that color diversity is driven by sexual selection^10^. This phenomenon may indicate a pivotal role of coloration in conveying sexual information to individuals of the same and opposite sex.

The role of colors may vary between sexes. For instance, in male lizards color conspicuousness has been associated with social dominance and intimidation of rivals^14–16^. Advertisement to mates is another potential function of male color conspicuousness since females have been documented to show preferences for brightly colored males^17,18^. Color signalling by females has been less often addressed in the literature, but some studies indicate gradients of ventral coloration as a possible fecundity/receptivity indicator and color polymorphism as associated with alternative reproductive strategies^19–22^. The association of sexual chromatic differences of certain body regions may help to understand imposed sexual selective pressures and the possible specific functions of the chromatic differences^23,24^.

In some families of lizards, the color differences between sexes seem to be more striking in areas of the body that can be viewed more easily by conspecifics than by predators. The chromatic diversity of these regions may be used in typical sexual selection contexts for advertising individual quality or status both intersexually^21,25,26^ and intrasexually^10^; and may enhance social displays^27^. The ventral and lateral regions of lizards are intuitively considered as informative to conspecifics, for both males^28–30^ and females^19,22^, whereas the dorsal regions are often duller in coloration and may show cryptic patterns in both sexes as a means of avoiding being sighted by avian predators. However, in some species dimorphism in color encompasses the whole body^31^; and dorsal coloration may be used to signal social status^32^.

The signalling function of sexually dimorphic coloration seems obvious if conspicuous patches are restricted to regions of the body that are only visible during certain displays, e.g. in contests with rival males or when males court females^33^. Lizards show a great wealth of bodily expressions to interact with one another^34,35^. The areas involved in displays may be subject to sexual selection, and color may be a key trait in strengthening the message transmitted through a display^34^. Hence, elucidating associations between color characteristics and body regions involved in chromatic communication may contribute to reveal the selective pressures acting on dichromatism.

Animal coloration should not be analysed with tools designed for human vision, as many animal species perceives colors in a radically different way^10^. A starting point to obtain objective reflectance measurements is spectrophotometric data, which accurately describes the magnitude of light reflected at each wavelength by a surface^36^.

Still, visual modelling is necessary to understand how the receivers of chromatic signals perceive their conspecifics colorations^37–39^ and how selective pressures are responsible for shaping chromatic communication^40–42^. Diurnal lizards in particular have four types of photopigments, one which is sensitive to near UV wavelengths^10,43,44^. The role of UV signals in intraspecific communication has been identified in a number of lizard species, in both males^25,26,45^ and females^19^. Nonetheless, whether UV signals are related to differences in coloration of lizard body regions is practically unknown.

Sexual dichromatism may be present during the entire year although sexual coloration may be enhanced during the reproductive season^46,47^. In some species, male color variability in the non breeding season may even have an important social signalling role. Sexual dichromatism may regulate social interactions that can give males a direct advantage during the next reproductive season (e.g. males agonistic interactions, securing a territory, attracting females, etc.). For instance, UV chroma signals territorial dominance and mediates aggressive behaviour in unrelated individuals in the non reproductive season in *Podarcis muralis*^48^ and in *Psammodromus algirus*, with permanent UV signatures indicating male quality and survival capacity^49^. Therefore, studying the expression of dichromatism in the non-breeding season may provide the basis of the role of dichromatism in conspecific communication.

**Figure 1:**
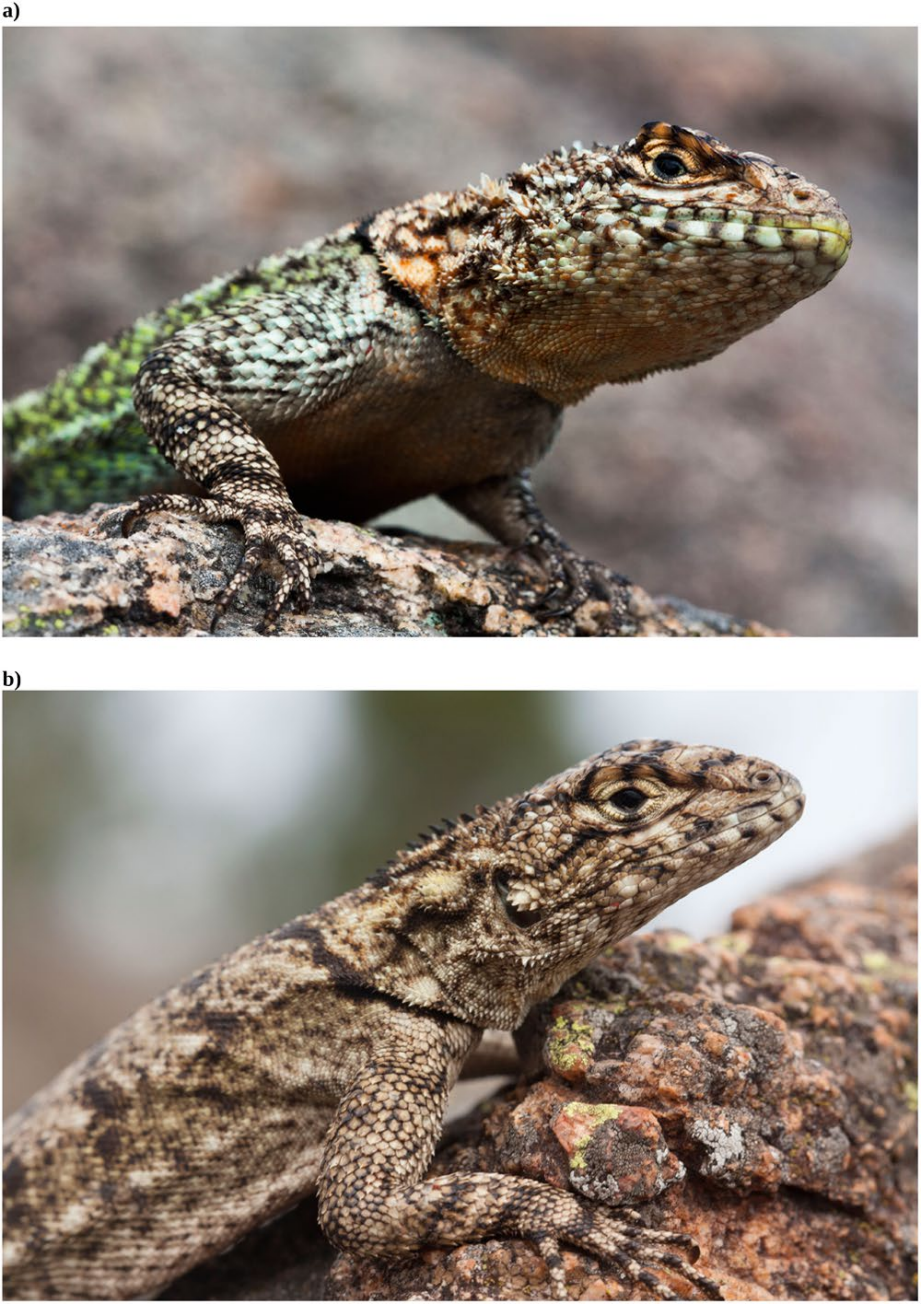
Male (**a**) and female (**b**) of *Tropidurus spinulosus*.

The family Tropiduridae belongs to the Iguaninae infraorder and includes animals traditionally regarded as highly “visual”^43^. *Tropidurus spinulosus* is among the most dimorphic species of the family^13^. Sexual selection is expected to have favoured dichromatism and color diversity biased to male coloration. Indeed, males exhibit a variety of different colors arranged in complex patterns (Fig. 1a), while female coloration is less striking (Fig. 1b). Moreover, *T*. *spinulosus* is a highly social species that performs interesting displays involving multiple regions of the body in which color expression is expected^50^. However, little is known about how their absolute spectral reflectance is visualized under a lizard visual system. The hypothesis formulated is that sexual selection through visual communication has driven sexual color differentiation by increasing male conspicuousness in *T*. *spinulosus*. Accordingly, it is expected that male biased dichromatism as well as male-biased color diversity will be detected, and, particularly, that chromatic differences between sexes will be concentrated in body regions used for visual displays in intraspecific communication. Consequently, the aim of this work was to provide a comprehensive picture of the dichromatism and color diversity of *T*. *spinulosus* by comparing sexes and body regions using lizard visual modelling.

## Results

*T*. *spinulosus* females and males showed significant chromatic differences (dS^51^) to conspecifics mainly on the ventral and flank regions, where cluster analysis isolated male specific clusters; significant values in achromatic differences (dL^51^) were found only on the abdomen and chest regions (Table 1; Supplementary Fig. S1). All perceived color contrasts caused by male coloration showed a Just Noticeable Differences (JND) value > 2, and on the abdomen and flanks it was even higher than 5.

**Table 1:** Sexual dichromatism for each body region by cluster analysis in *Tropidurus spinulosus*.

	Tree clust	dS				dL			
		JND	%tot	%within	%males	JND	%tot	%within	%males
Abdomen	2	5.91	47	100	100	3.87	20	95	100
Chest	3	3	10	100	53	2.13	39	97	58
Cloaca	3	2.49	24	100	100	1.89	14	75	0
Dorsum	0	3.69	7	89	0	2.80	2	54	0
Flanks	2	5.53	11	100	100	2.03	3	50	0
Head	0	3.88	6	57	0	5.60	9.60	63	0
Limbs	0	3.16	4.2	68	0	1.93	11.55	67.30	0
Mouth	0	3.72	0.6	100	0	3.46	2	63	0
Head profile	0	7.76	0.8	100	0	3.30	3.60	50	0
Throat	2	4.89	42	95	100	2.46	3.70	75	0

The final tree with the total number of clusters where dichromatism was found is reported in the column “Tree clust”; in all body regions only one cluster was dichromatic, and male-specific. Those regions that did not show dichromatism were classified as 0. Columns “JND”, “%tot” and “%within” stand for the three criteria for a cluster to be considered numerically relevant within the region and sex specific: “JND” is the mean difference in JND between the significant cluster and the rest of the clusters of the tree, which should be higher than 2 to be considered visually discernible. “%tot” represents the percentage of the sampled points included in the significant cluster considering the total for the region of interest, which should be higher than 10%. “%within” represents the internal composition of the significant cluster and the percentage of points belonging to one of the two sexes, and should be higher than 90%. “%males” indicates the percentage of males that contributed with their spectra to the significant cluster.

The remaining body regions (Table 1; Supplementary Fig. S1) presented clusters shared by both sexes. We assumed that these regions are not perceived as sexually dichromatic.

Males scored significantly higher in all the color diversity indices in all the regions that showed dichromatism; moreover significant differences between sexes were found even for Dorsum and Profhead (Table 2).

**Table 2:** Color diversity and UV indices of the tetrahedrons of males and females.

	Color volume		Hue disparity mean		Hue disparity var		Color span mean		Color span var		UV.centroid	
Region	Female	Male	Female	Male	Female	Male	Female	Male	Female	Male	Female	Male
Abdomen	1.19E-06	**3.32E-05**	7.54E-02	**1.55E-01**	2.85E-03	**1.07E-02**	1.38E-02	**3.91E-02**	5.25E-05	**5.53E-04**	**1.32E-01**	7.00E-02
Chest	9.17E-08	**1.88E-06**	4.54E-02	**1.77E-01**	9.20E-04	**1.68E-02**	8.94E-03	**3.46E-02**	2.12E-05	**5.44E-04**	**1.37E-01**	1.26E-01
Cloaca	2.15E-06	**1.67E-05**	1.10E-01	**1.60E-01**	5.85E-03	**1.09E-02**	1.75E-02	**3.65E-02**	9.56E-05	**5.03E-04**	**1.34E-01**	1.12E-01
Dorsum	6.39E-06	**3.29E-05**	1.29E-01	**2.07E-01**	1.34E-02	2.36E-02	2.35E-02	**3.93E-02**	2.30E-04	**7.46E-04**	1.38E-01	1.42E-01
Flanks	2.41E-05	**1.45E-04**	2.12E-01	**2.52E-01**	3.72E-02	3.14E-02	2.81E-02	**5.02E-02**	4.30E-04	**9.81E-04**	1.49E-01	1.35E-01
Head	1.84E-05	2.16E-05	2.08E-01	2.06E-01	9.37E-02	4.58E-02	3.80E-02	4.26E-02	1.24E-03	8.49E-04	1.16E-01	1.14E-01
Limbs	6.06E-06	8.52E-06	2.18E-01	2.15E-01	7.18E-02	5.85E-02	3.05E-02	3.07E-02	6.46E-04	5.18E-04	1.46E-01	1.46E-01
Mouth	2.47E-06	5.77E-06	2.23E-01	2.01E-01	3.63E-02	2.08E-02	2.55E-02	2.83E-02	2.88E-04	3.43E-04	1.68E-01	1.68E-01
Head profile (Profhead)	2.68E-05	**5.53E-05**	2.44E-01	2.71E-01	6.08E-02	5.36E-02	4.00E-02	4.62E-02	6.58E-04	9.74E-04	1.39E-01	1.36E-01
Throat	2.42E-06	**1.11E-05**	9.55E-02	**1.32E-01**	4.51E-03	**8.63E-03**	1.92E-02	**3.39E-02**	1.25E-04	**4.15E-04**	**1.16E-01**	6.93E-02

Values highlighted in bold represent the sex with a higher and statistically significant mean (randomization procedure). The “uv.centroid” reflects the relative stimulation of the UV cones of N.sagrei visual system. Tetrahedrons color volume (“rel.c.vol”), color span mean and variance (“colspan.m”;“colspan.v”) and Hue disparity mean and variance (“huedisp.m”; “huedisp.v”) follow the Stoddard and Plum (2008) procedure.

**Figure 2:**
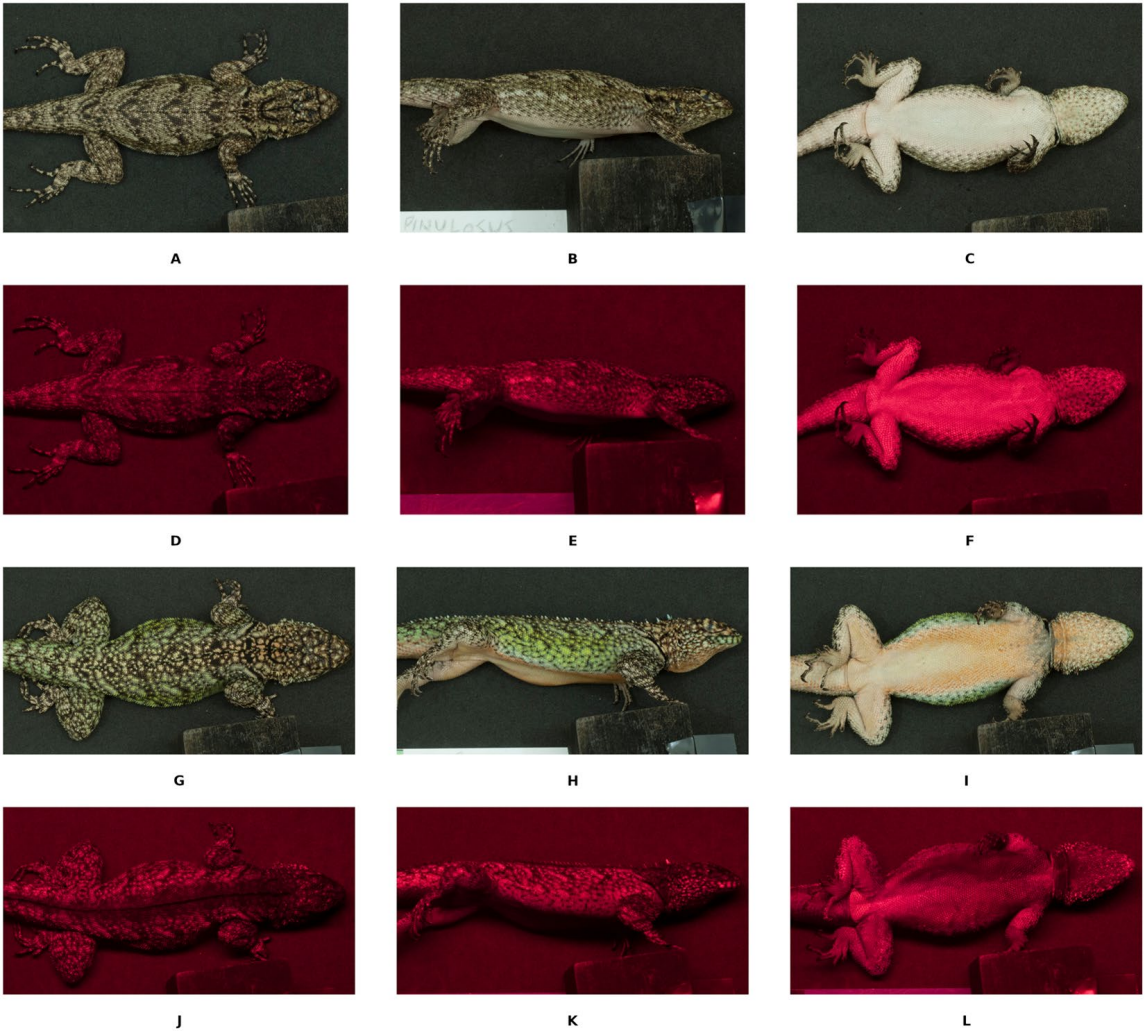
Pictures of *T. spinulosus* showing visible and ultraviolet wavelengths for each of the three regions (dorsal, lateral, ventral) for both females (visible: **A**–**C**; ultraviolet: **D**–**F**) and males (visible: **G**–**I**; ultraviolet: **J**–**L**). Pictures were taken after the spectrophotometric measurements, lizards were put in plastic containers for 10 minutes to acclimate at room temperature (25 °C). A Nikon D70 equipped with a Nikon 60 mm macro lens mounted on a tripod was used to take visible pictures, to which a B + W 403 UV-pass filter was fitted to take UV pictures. Lizards were placed in a plastic container with the surface covered by black poly-ethilvinilacetate, a spectrally flat material; photos were taken in a dark room to avoid infrared contamination. Light sources for visible photographs were provided by two led lamps (Sica bulb 7 W) mounted on tripods and pointed at 130° towards the ceiling and away from the subject to avoid burnt pixels. A black UV-A tube (Blacklite Fluorescent Fixture) was used as a UV source. As the tube had low UV output we decided to point it directly towards the subject; UV irradiation was brief and did not harm the animals involved in the study. Visible-wavelength pictures were taken at ISO 400, 2 seconds shutter speed and f-16; whereas UV pictures were taken at ISO 400, 15 seconds shutter speed and f-16. Pictures from the camera were cropped with the open source software GIMP^77^.

The ventral regions of females produced higher stimulation of the UV cone according to the visual system used (Table 2, see “centroid.uv”). On the throat, chest and abdomen, UV-poor wavelength colors (orange to red) characterised male coloration, whereas UV-rich white coloration characterised female coloration (Fig. 2C,F,I,L; Cluster 2 of Abdomen and Throat, Supplementary Fig. S3). Accordingly, ventral regions of males were significantly darker (even with absolute black scales in the chest) than those of females (dL plots for Abdomen, Chest and Throat, Supplementary Fig. S1; Fig. 2C,F,I,L). However, some males did have patches similar to those of females that were grouped together in a female-biased cluster (dS plots for Abdomen and Throat, Supplementary Fig. S1).

On the cloacal region, male-specific clusters showed a more yellowish hue, while a shared cluster included UV-rich white coloration (Fig. 2C,F,I,L; Cluster 2 of Cloaca, Supplementary Fig. S3). On the flanks, the male cluster was mainly composed of UV-poor blue located in the outer ventral scales, green scales and yellow scales (Fig. 2H–K; Cluster 2 of the Flanks, Supplementary Fig. S3). A few males showed a faint UV trace on the mouth region, which was probably masked in the pooled analysis together with males that showed no UV trace (Fig. 2B,E,H,K).

## Discussion

This work contributes to the understanding of dichromatism in *T*. *spinulosus* by elucidating associations between color characteristics and body regions in both sexes, suggesting that sexual selective pressures are operating through chromatic communication. Our results show that *T*. *spinulosus* is perceived by conspecifics as sexually dichromatic, and that conspecifics perceive a wider range of colors (larger color volumes) with higher contrast (hue disparity) and chroma (color span) in males than in females. Dichromatism is expressed in regions that are traditionally linked to intraspecific communication^9,10,52^.

On the flank region, the dichromatism perceived under the visual model is mainly due to male specific UV-poor light blue spectra reflected by the outer ventral scales together with medium-wavelength (green) and long-wavelength yellow type spectra. In the phylogenetically close species *Crotatphytus collaris*, a similar type of light blue pigmentation causes the highest chromatic dimorphism between sexes^31^. In other species, short-wavelength spots on the flanks are commonly associated with the presence of UV-components during the non breeding season (*Podarcis muralis*^48^*; Psammodromus algirus*^49^) and are also important signals to conspecifics in the breeding season (*Podarcis muralis*^30^ and *Lacerta viridis*^26^). Moreover, flanks are arguably one of the most visible regions during behavioural displays. In *T*. *spinulosus* the color patches on the lateral side are presented to the opponent during the *circulation display*, where both males move in circles showing the lateral sides to each other while simultaneously performing a *gaping display* (Perez 1988^50^; Rossi N., pers. Obs.).

The role of green coloration can be ambiguous as in some species it is used as nuptial coloration^32,53,54^ while in others its biological importance is not clear or, simply, green coloration could serve as contrast with yellow pigmentation^55^. In *T*. *spinulosus* green scales are spatially close to yellow scales constituting a pattern similar to the nuptial coloration of *Lacerta viridis*^56^. The extension of yellow pigmentation on the flanks has been correlated with indices of sexual selection in Agamidae^57^.

Our results suggest that dichromatism is often associated with color diversity in the study species supporting the prediction that sexual selection may have acted on the complexity of males phenotypes^9^. However, we also detected significant differences in color volume between sexes in the dorsum and the side of the head regions that did not express JND differences. The latter is visually exposed during displays such as *push-ups* and *headbobs*, and its color diversity may be associated with residual coloration from the breeding season^58^. On the other hand, on the dorsal region the diversity of colors may be important in agonistic behaviours, as was found in *Tropidurus semitaeniatus* which also exhibits complex dorsal color patterns that may convey individual quality information^59^.

*Tropidurus spinulosus* female coloration was found to be strongly UV-biased and brighter than males on the abdominal regions, which causes a high achromatic contrast with respect to male coloration. Female UV patterns were also found in other species^19,31^ such as *Ctenophorus ornatus*, where UV-chroma correlates with the sexual receptivity of females and is actively selected by males^19^. In other species carotenoid-based colors usually signal female reproductive status^20–22,60^ and, in some cases, UV-rich white (or grey) is the “baseline coloration” to which red coloration fades in the non breeding period (e.g. *Acanthodactylus erythrurus*^61^). In female *T*. *spinulosus* no patch reflects singularly in the UV, suggesting that the absence of UV-absorbing pigments may cause UV reflectance^62^. Similarly, in males, some ventral scales that are presumably devoid of pigment are grouped together with female scales while those that do have pigments cause the male-specific, orange cluster. Pigmentation of the ventral regions has been associated with male dominance in some lizard species^30,63,64^ and, in *T*. *spinulosus*, is shown during males’ *push-ups*, performed in ritual courtship^65^ and combat (NR, unpublished data). Besides the female biased UV dichromatism we found, we also measured faint UV traces associated with green colors on the labial scales of some males; these traces could prove interesting, since males perform a *gaping display* during male ritual combat and UV mouth coloration is associated with male fighting ability in other species^31,66^. On the cloacal region, the male cluster is characterised by a yellow hue that is also found in fellows Tropidurid lizard, especially in *Tropidurus semitaeniatus* during the non-breeding season, which shifts to black during the breeding season depending on male size^67^. coloration of the cloacal region of females shifts from white to orange/red during breeding season (NR, unpublished data). The cloaca is shown to conspecifics during the *tail whipping* display in females during courtship/copulation^65^ and in males during male-male interactions (NR, unpublished data).

Both male and female coloration should be further assessed during the breeding season, since coloration may change to enhance the baseline coloration and boost color diversity in relation to changes in reproductive condition, expressing *dynamic dichromatism*. Chroma or brilliance of colors, and the area of color on body regions change in some species, especially in males, to signal competitive ability^53^. Color contrasts can be further intensified through a color darkening mechanism, an ability that is present in our study species (unpublished data, NR) and that also affects mate choice dynamics in other species^32^. Elucidating the link between color variability, social dynamics and sex-specific reproductive parameters and the relationship of these with the displays expressed in each body region will make an important contribution to understanding the role of phenotypic variability in the context of sexual selection.

In conclusion, the results show that dichromatism and color diversity under a conspecific visual model are male biased in this study species supporting the hypothesis that sexual selection may be responsible for differences in male and female phenotypes. Also, both dichromatism and differences in color diversity occur mainly on the ventral and flank regions, which traditionally have been closely linked to social interactions.

## Methods

### Study species and spectral measurements

*Tropidurus spinulosus* is among the most male-biased dimorphic species in body size within the Chaco region of South America, with males being 16% larger than females (Males SVL = 112.62 ± 9.51 mm, Females SVL = 96.58 ± 7.15 mm; Sexual Size Dimorphic Index, SSD = 0.22). Moreover, male-biased sexual dimorphism is evident in the size of certain body regions, such as the head^13^. This species is active most of the year. The study population is located in the province of Córdoba, central Argentina. Permission for scientific capture was granted by the local government environmental office (Secretaría de Ambiente y Cambio Climático – permit number: 546833053717).

Adult specimens (*n* males = 13, *n* females = 12) were captured in autumn by noosing, outside the species reproductive period. Geographic coordinates of the exact capture site were recorded with a GPS (Garmin eTrek 30). In the laboratory, lizards were kept individually in plastic containers under fixed light (9–17 hs, Zoomed UVB 5.0 UV tubes) and temperature (25 °C); larvae of *Tenebrio molitor* and water were provided *ad libitum*. Spectrophotometric measurements were taken within a week of capture. Specimens showing signs of ongoing moulting were not sampled, because colors become duller during that process. After the measurements were taken, all subjects were released at their original site of capture.

Spectral data were obtained using an Ocean Optics USB4000 (Ocean Optics, 830 Douglas Ave., Dunedin, FL, USA 34698) spectrophotometer coupled with a halogen and deuterium light source, both connected to the sensor by a bifurcated fibre optic cable. The probe was inserted into a rectangular prism holder at 45° to avoid specular reflections and the head of the probe was placed at the bottom of the prism at an approximate distance of 4 mm from the sampled surface. This distance corresponds to a reading area of 2.36 mm^2,68^, which was enough to measure small color patches accurately (2.5 mm on average), but not single scales (1 mm long on average measured with ImageJ)^69^. Reflectance was measured relative to a white standard (Ocean Optics, WS-1-SS White Standard) and dark standard (lamps switched off and probe covered); both standards were periodically reset to account for fluctuations in the environment.

### Sampling design

Male *T*. *spinulosus* lizards exhibit a complex pattern of small patches with different colors and hues, whilst female patterns are less striking. An exploratory sampling was carried out, consisting in taking a large number of samples per region (n = 60 reflectance spectra). Ten body regions involved in intraspecific displays were selected (Table 3). To determine the final number of samples to take for each body region, the reflectance values of the wavelength with the most variability were plotted against a number of samples and the onset of a plateau was visually determined (the final number of spectra covered all the color variability inside body areas as shown in Table 3).

**Table 3:** Sampled body regions and their role in conspecific communication.

Body region	Rationale	Displays in *T*. *spinulosus*	N° spectra taken
Abdomen	Ventral coloration is important in many Squamata species to convey sexual signals, e.g. in females it may indicate reproductive status^22^ and in males the extension of color patches may be used as an indicator of male quality^16,78^	Push-ups: males show proximal ventral regions when patrolling their territory (NR, unpublished data)	16
Chest	In some species it can be used to convey sexual signals, and is shown during pushup displays^19,52^		8
Throat	Throat coloration is used to convey sexual signals in many species, both in females^21^ and males^25,26^		16
Cloaca	The cloacal region is involved in some sexual behaviours (licking^79,80^), e.g. in *T*. *semitaeniatus* cloacal pigmentation correlates with sperm production in males^81^	Tail whipping: Shown by females during courtship^65^ and sometimes in male-male interactions (NR, unpublished data)	16
Dorsum	In some species, male dorsal coloration may provide social status signals^32,82^. *Tropidurus semitaeniatus* performs a *dorsum display* during combat^59^	No displays in *T*. *spinulosus* were observed for this region.	42
Flanks	Together with the ventral region, this is one of the most important parts in lizard sexual and social communication^10,30,52^	*Circulation display:* Shown by male during male ritual combat (^50^; NR, unpublished data). *Lateral approach:* male approach to females at the beginning of the courtship^65^.	84
Head	Reproductive coloration extends to this region in males of some species^83^.	No displays in *T*. *spinulosus* were observed for this region.	20
Legs	Many lizard species use legs to communicate (waving^35^).	No displays in *T*. *spinulosus* were observed for this region.	16
Mouth	This can be used as an “advertisement” for male bite force^75^. In *C*. *collaris* dominant males show stronger UV signature in this region^84^.	*Gaping display:* performed during male ritual combat (NR unpublished data).	10
Head Profile (Profhead)	Male nuptial coloration is shown in this region and it is clearly visible in many displays^52,85^. In *T*. *spinulosus* the coloration shifts from whitish to orange during the reproductive season^86^.	*Push-ups*, *headbobs*, *circulation* (NR, unpublished data), *lateral approach*^64^^.^	20

“N° spectra taken” reports the final number of spectra taken in each body region for each individual.

### Spectra processing and visual modelling

Spectra were imported into R software^70^ and processed with the *pavo package*^51^, which provides practical tools to implement visual systems in an R environment. Spectra were LOESS smoothed (span = 0.2, *procspec* function) to avoid unwanted noise in reflectance spectra. To analyse color differences according to how these are perceived by animals, a visual model which considers relative cone excitation of the lizard visual system was applied. Visual model parameters were defined following Fleishman, 2016^55^, although the *sensemodel* quantum catches function was calculated under “bluesky” conditions, because *T*. *spinulosus* lives in rocky outcrops where it receives direct sunlight and the background was set as “ideal” (function *vismodel*). In lizards, visual systems seem to be phylogenetically conserved^43^: therefore, we used the cone sensitivities of *Norops sagrei*^44^, a species belonging to the same *Pleurodonta/Iguanoidea* clade as *Tropidurus*^44^ and showing coloration under similar full sun condition to those of the focal species^44^.

Chromatic (dS) and achromatic (dL) distances were calculated in Just Noticeable Differences units (JND following the receptor-noise model^71^, using the function *coldist*. This model assumes that color discrimination is limited by photoreceptor noise. Thus, color distances are calculated by using the inverse of the noise-to-signal ratio, known as the Weber fraction, which depends on the noise-to-signal ratio of each cone and the relative number of receptor cells of types. Visual stimuli separated by one JND are theoretically discernible by the lizard eye, although many publications have adopted a more conservative threshold of at least two JND^43,72^. dS and dL were calculated between all the points sampled within a body region without accounting for sex or individual. To our knowledge, there is no information available on *N*. *sagrei* cone proportions or Weber fraction (and very little in lizards in general). Therefore, JNDs with multiple combinations of cone proportions were calculated to avoid biased results^68^: (1) a cone proportion that emphasizes the long-wavelength sensitive opsins (LWS hereafter^55^); (2) one that sets an equal proportions of cones^41^; (3) one that proposes different weights for dS and dL, because, in some species, double cones (putatively responsible for light perception) are more abundant than the other cones^73^. Cone proportions did not affect the results of the cluster analysis; the results shown below are derived from cone proportions reported in Fleishman and collaborators 2016^55^.

The relative quantum catches stimulation was used to convert spectra into coordinates of the tetrahedral color space (function *colspace*). color loci were obtained for each combination of body region and individual and then color diversity indices were extracted following procedures reported by Stoddard and Prum, 2008^74^ (function *summary*.*colspace*): *color volume* measures the colors diversity of a given body region; *hue disparity* mean and variance describe the mean and variance in color contrasts within the tetrahedron only in terms of hue, while *color span*, which is the mean Euclidean distance between the points in the tetrahedron, also takes chroma into account. In addition, we calculated the *mean stimulation for the ultraviolet sensitive opsins* (*UVS*) *cone* for each body region to test for cryptic dichromatism.

### Statistical analysis

Sexual dichromatism is often assessed as differences in color between sexes in a specific body region^75^. However, *T*. *spinulosus* exhibits complex coloration patterns consisting of many small patches with different colors, and thus we expect that dichromatism may arise by the presence of a given color patch in one sex but not in the other. To test sexual dichromatism, we first needed a procedure that helps to identify different color patches in a body region, without any *a priori* knowledge. We applied a hierarchical cluster analysis on dS and dL distances. Clustering methods are unsupervised learning techniques that reveal homogeneous subgroups or clusters in a data set. In this way, we recovered the diversity of color patches within each body region, allowing us to identify male- or female-exclusive color patches. Cluster analysis was applied for each body region with the *hclust* function and the agglomerative “average” linkage method (UPGMA). In the resulting trees, cuts were examined at five progressive heights, resulting in an increased number of clusters (2 to 6 clusters, function *cutree*). As our interest was to detect sexual dichromatism and not merely to identify clusters, a series of rules was followed instead of common pattern recognition algorithms. First, all the sample points forming clusters with only one observation were considered outliers and excluded from the analysis; the number of sample points excluded was not significant (n = 51, total n = 6500) and was equally distributed among body regions and sexes (data not reported). Second, the trees at each body region were inspected and sexual dichromatism was recognized if one or more clusters met these three criteria: (1) 90% of the observations within a cluster belonged to a single sex; (2) the cluster had at least 10% of the total observations for that body region: and (3) there was a minimum difference of 2 JND with the other clusters (we used this value because it is considered more conservative than the traditional JND = 1)^76^. Thus, for each body region a series of clusters was obtained. Each cluster represents homogeneous color patches that should cause the same type of stimulation to the lizard eye.

While cluster analysis can help to identify and describe distinguishable color patterns in a body region, sexual differences in coloration can also arise as a result of the simultaneous expression of different colors in a region. Therefore, it was assessed whether sexual differences in contrast and color diversity were significantly larger than expected by chance. To do this, the observed differences between sexes in color volume, hue disparity (mean and variance), colors span (see above) and the centroid of the UVS cone were compared with their random expectations obtained by randomly assigning sex to the color loci. Randomization was repeated 1000 times, and a given observed difference between male and female indices was considered significant when it was larger than the 95% percentile of the differences obtained by chance. All tests were conducted in R version 3.4.1^70^.

### Ethical approval

All applicable international, national, and/or institutional guidelines for the care and use of animals were followed. All procedures performed in studies involving animals were in accordance with the ethical standards of the institution or practice at which the studies were conducted. This research was approved by the Ethical Committee of the Instituto de Diversidad y Ecología Animal CONICET-UNC (protocol number: 2/2017).

## Supplementary information

Supplementary information

## Data Availability

The datasets generated during and/or analysed during the current study are available from the corresponding author on reasonable request.
