# Supplementary material for: Sexual dichromatism and color diversity in the spiny lava lizard Tropidurus spinulosus using lizard visual modelling
Source: Sci Rep. 2019 Oct 3;9:14270. doi: 10.1038/s41598-019-50712-0 (PMC6776660; doi:10.1038/s41598-019-50712-0)
Supplement: Supplementary file 1 — Supplementary information [file 41598_2019_50712_MOESM1_ESM.pdf]

Supplementary information

Manuscript title: Sexual dichromatism and color diversity in the spiny lava lizard *Tropidurus spinulosus* using lizard visual modelling.

Authors: Rossi N., Benitez-Vieyra S., Cocucci A., Chiaraviglio M., Cardozo G.

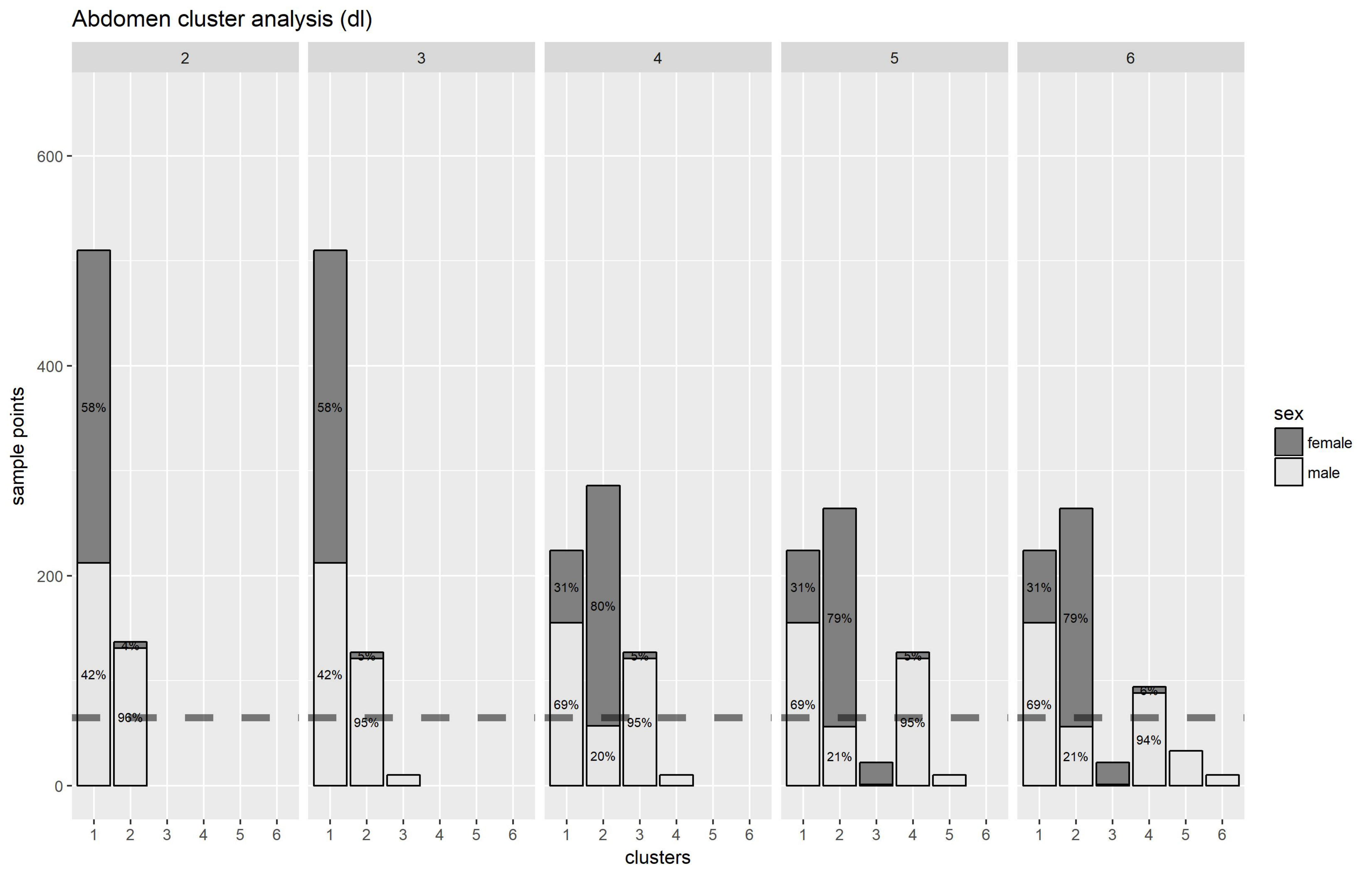
Abdomen cluster analysis (dl)
2
3
4
5
6
sample points
600
400
200
0
58%
42%
4%
96%
58%
42%
5%
95%
31%
69%
80%
20%
5%
95%
31%
69%
79%
21%
5%
95%
31%
69%
79%
21%
6%
94%
clusters
1
2
3
4
5
6
sex
female
male

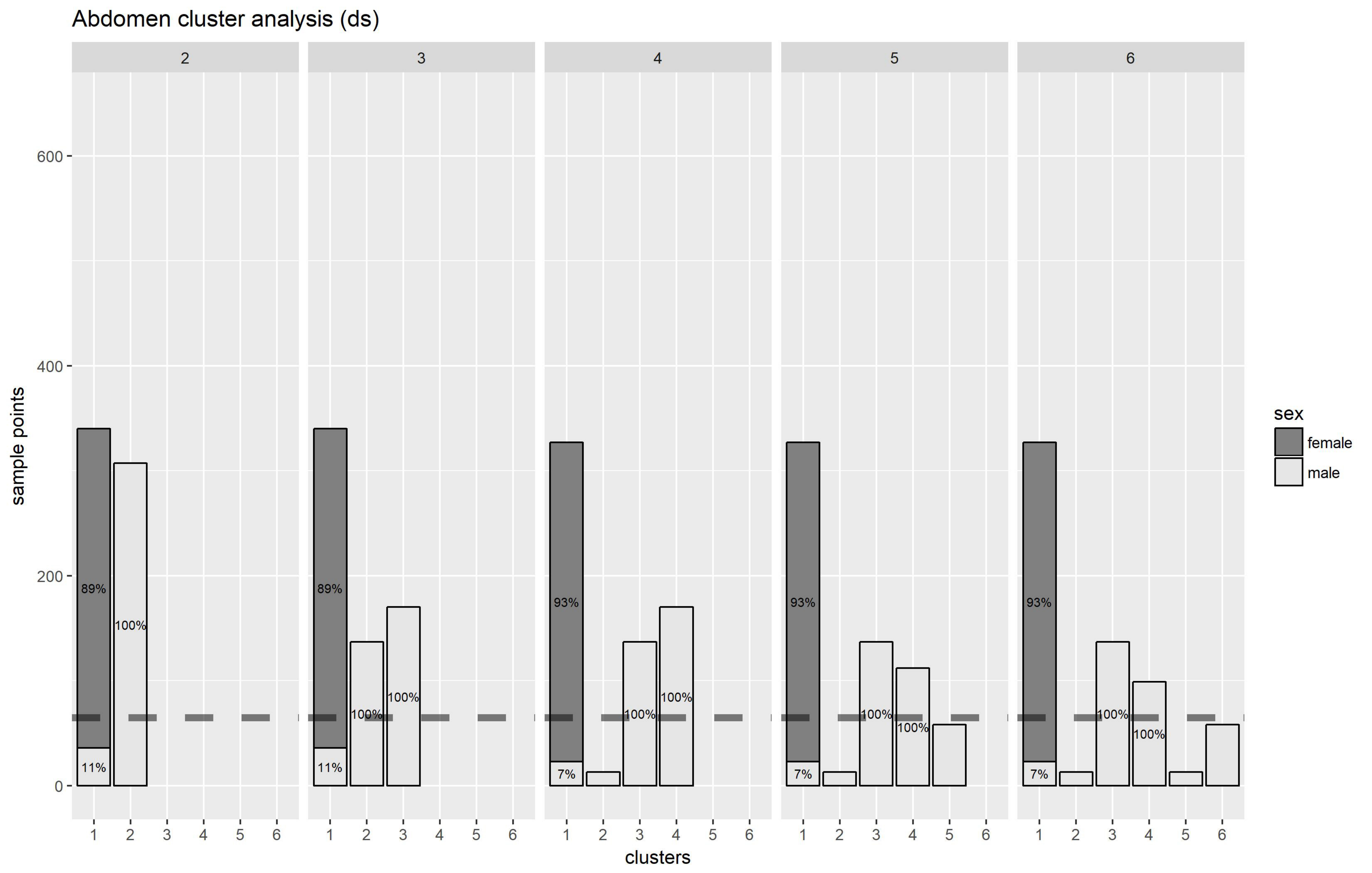

Abdomen cluster analysis (ds)
2
3
4
5
6
600
400
200
0
sample points
89%
100%
11%
89%
100%
100%
11%
93%
100%
100%
7%
93%
100%
100%
7%
93%
100%
100%
7%
1 2 3 4 5 6
clusters
sex
female
male

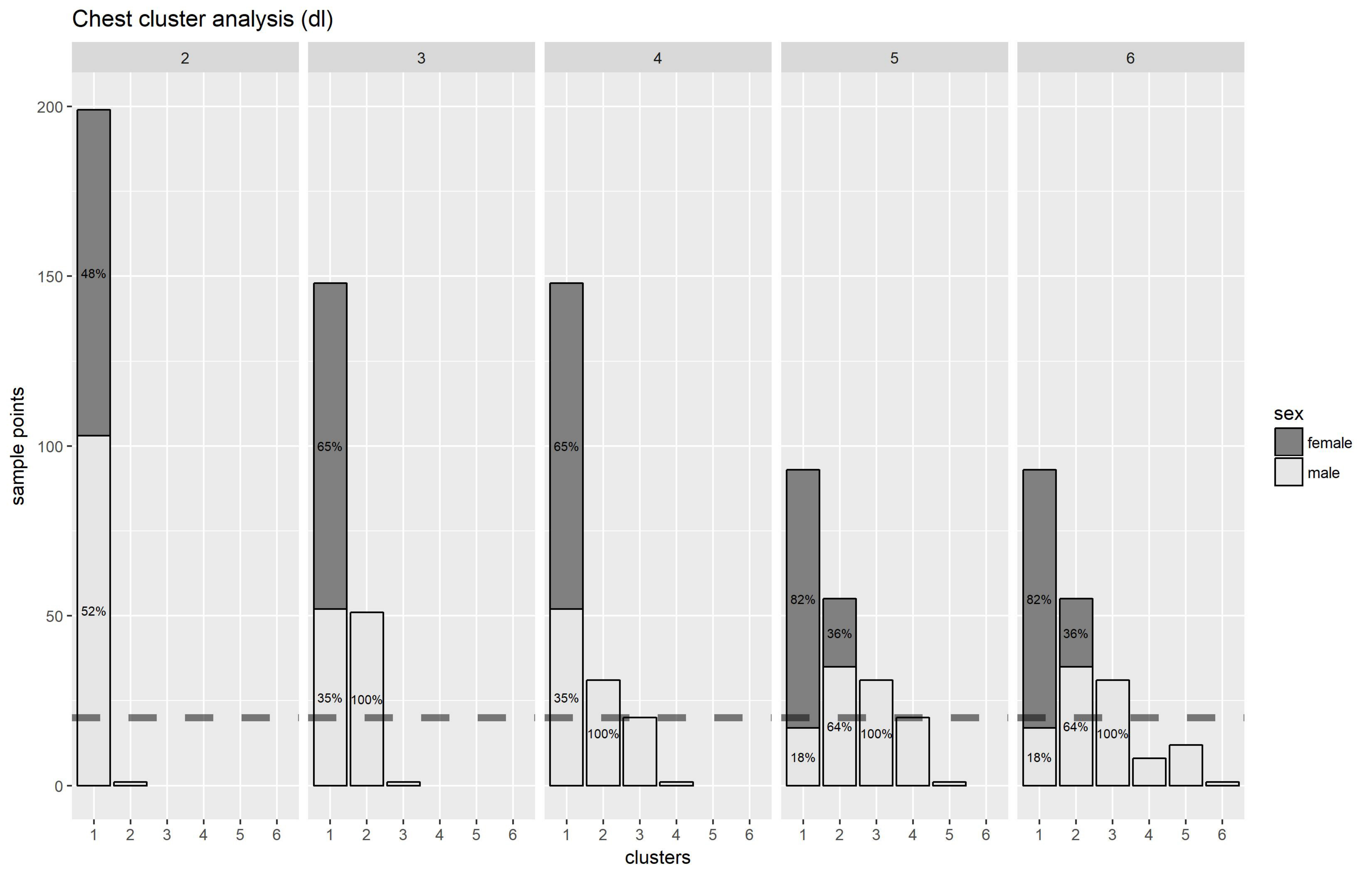
Chest cluster analysis (dl)
2
3
4
5
6
sample points
200
150
100
50
0
48%
52%
65%
35%
100%
82%
18%
36%
64%
clusters
1
2
3
4
5
6
sex
female
male

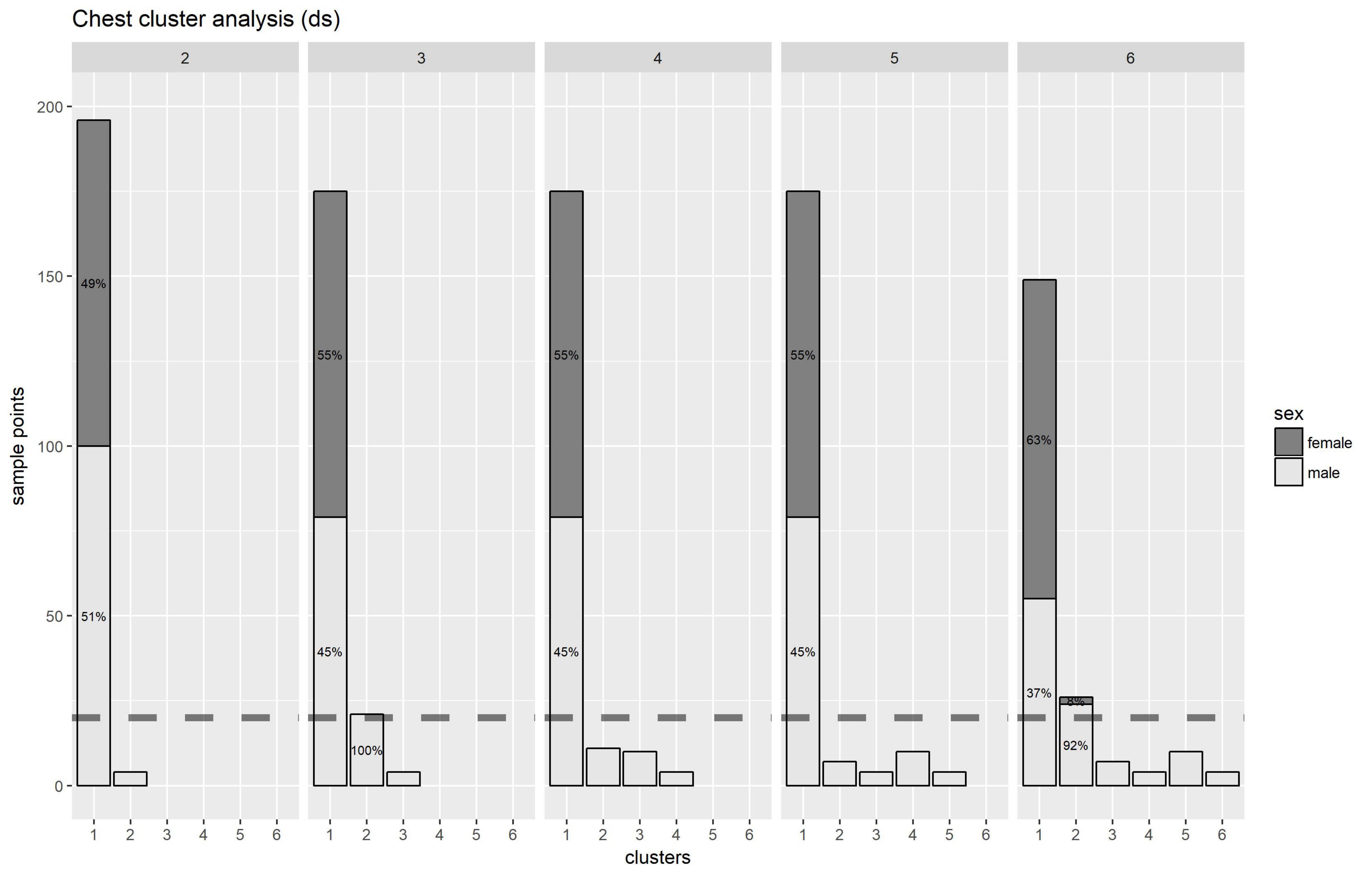
Chest cluster analysis (ds)
2
3
4
5
6
sample points
clusters
sex
female
male
49%
51%
55%
45%
100%
63%
37%
8%
92%
0
50
100
150
200
1
2
3
4
5
6

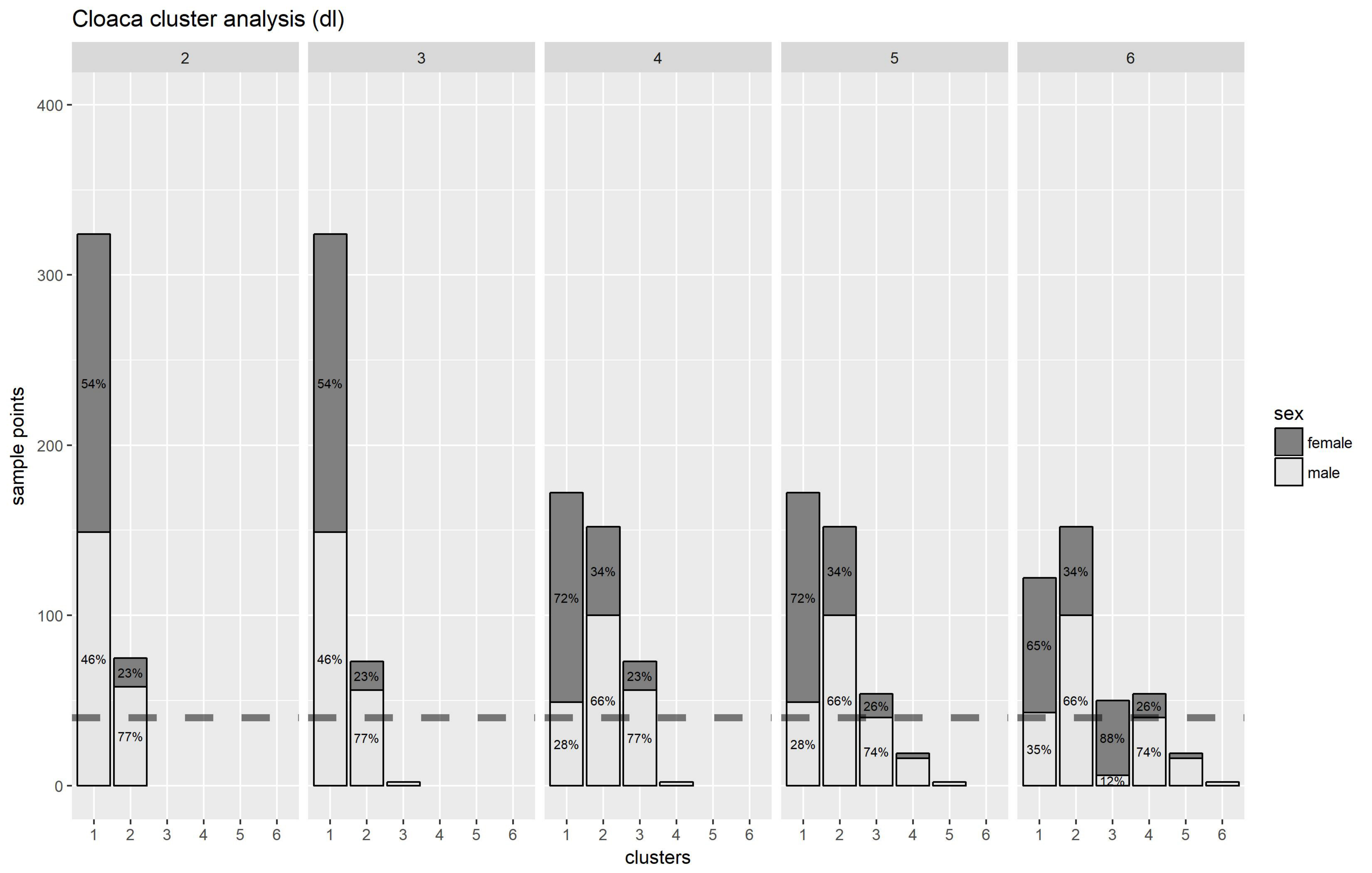
Cloaca cluster analysis (dl)
2
3
4
5
6
sample points
400
300
200
100
0
54%
46%
23%
77%
72%
28%
34%
66%
74%
26%
65%
35%
88%
12%
1
2
3
4
5
6
clusters
sex
female
male

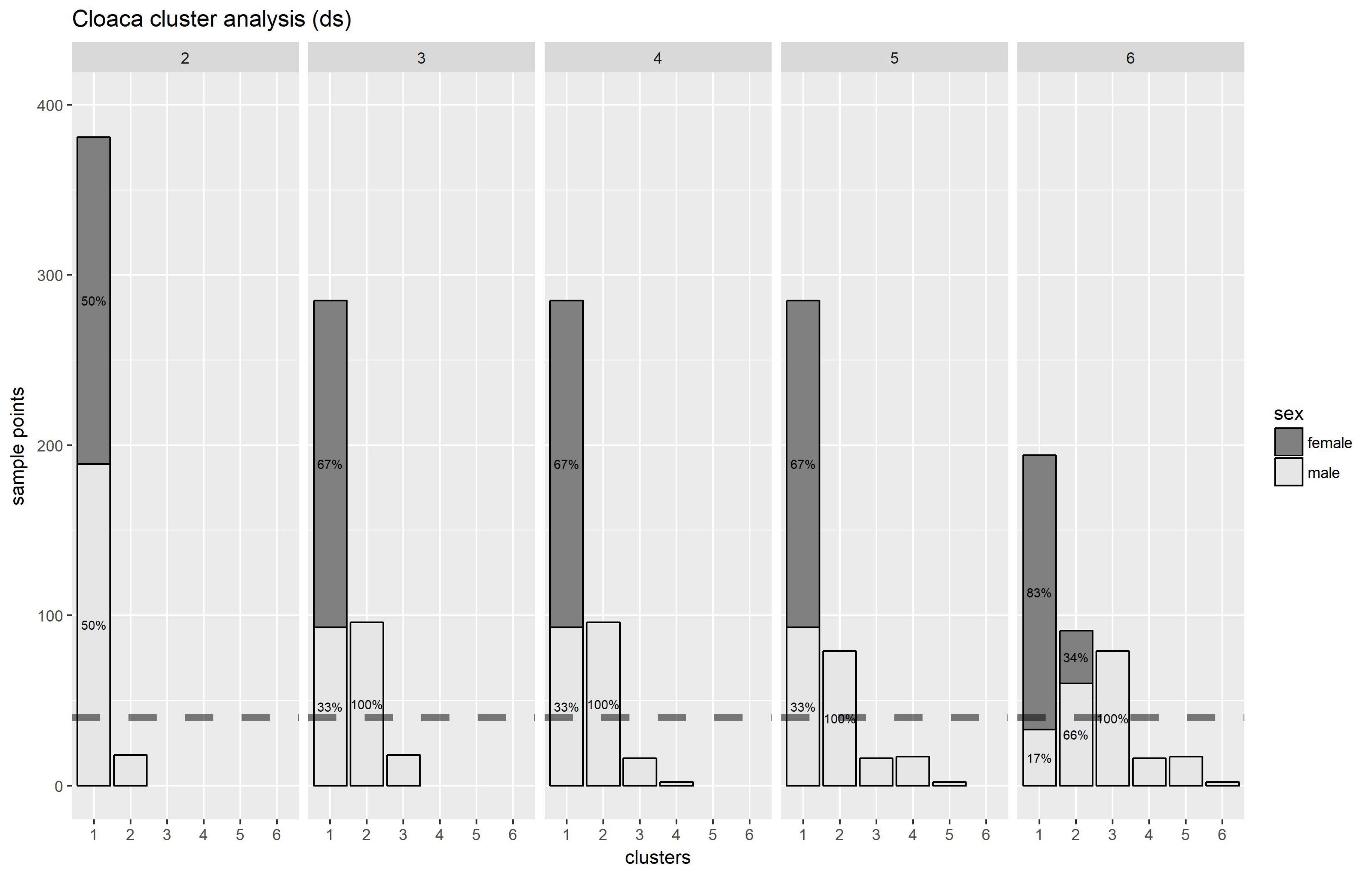

Cloaca cluster analysis (ds)
2
3
4
5
6
sample points
clusters
sex
female
male
0
100
200
300
400
1
2
3
4
5
6
50%
50%
67%
33%
100%
83%
34%
66%
17%

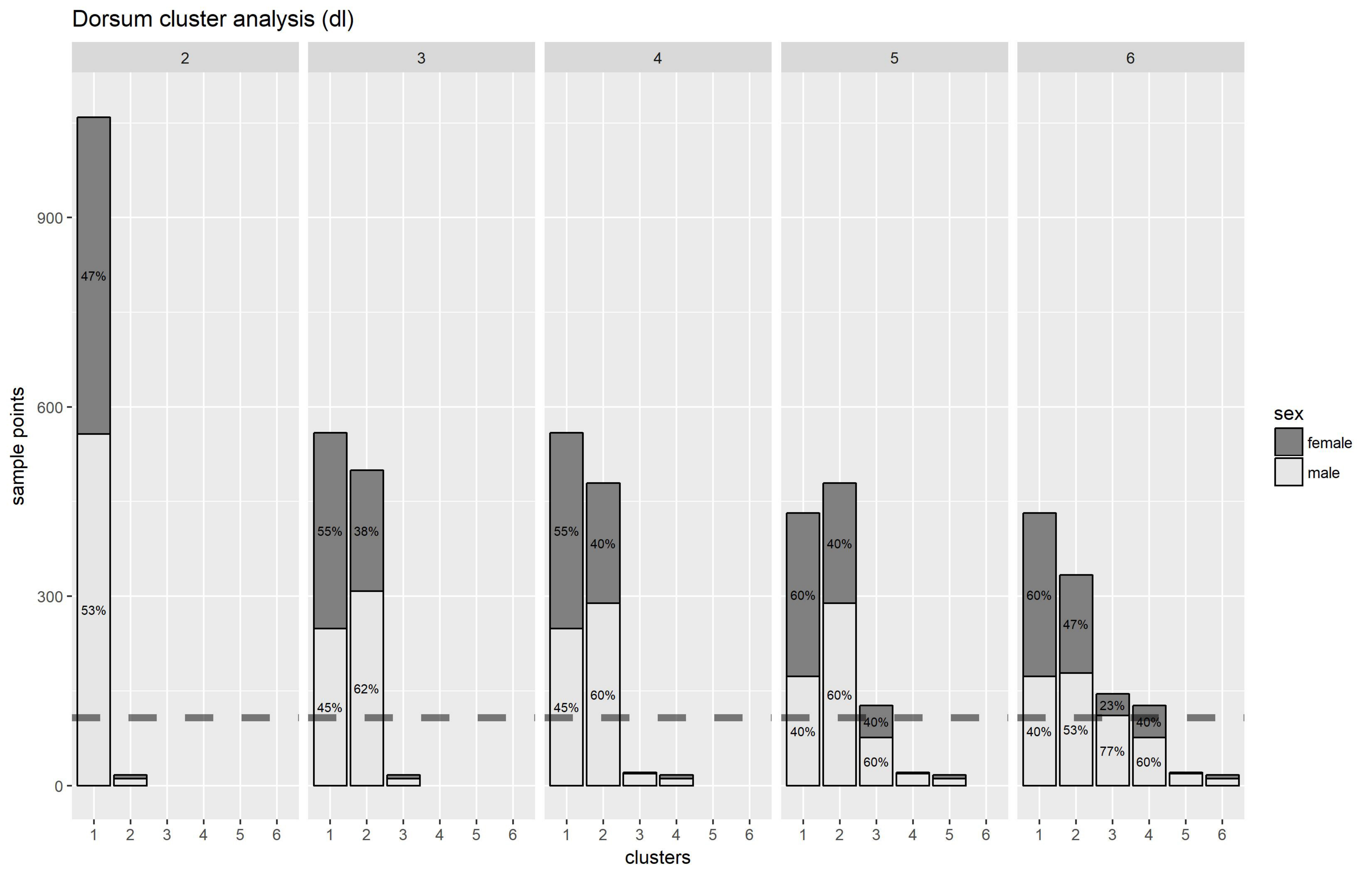

Dorsum cluster analysis (dl)
2
3
4
5
6
sample points
clusters
sex
female
male
900
600
300
0
1
2
3
4
5
6
47%
53%
55%
45%
38%
62%
55%
45%
40%
60%
60%
40%
40%
60%
40%
60%
60%
40%
47%
53%
23%
77%
40%
60%

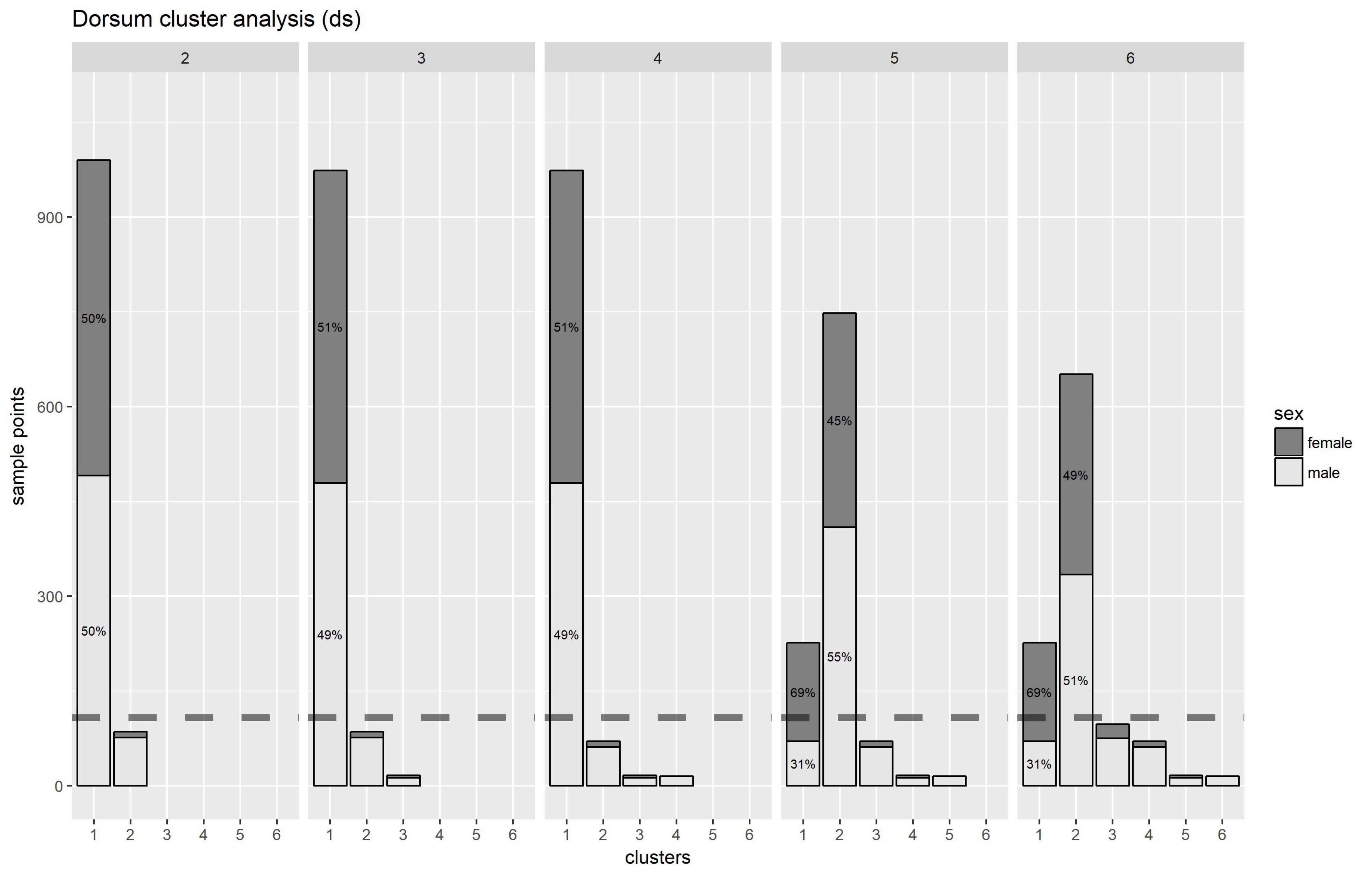
Dorsum cluster analysis (ds)
2
3
4
5
6
sample points
900
600
300
0
50%
50%
51%
49%
51%
49%
45%
55%
69%
31%
49%
51%
69%
31%
1
2
3
4
5
6
clusters
sex
female
male

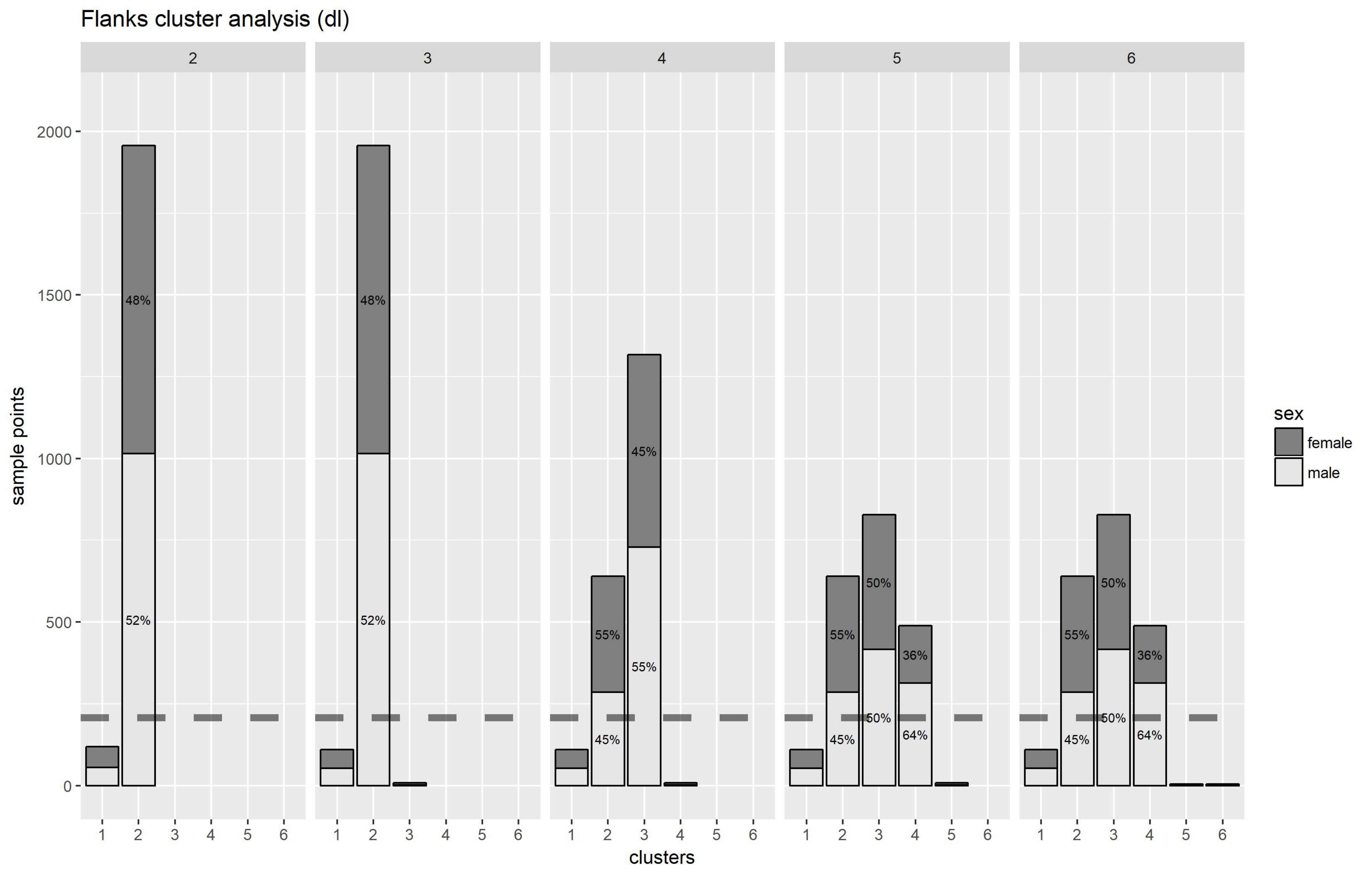

Flanks cluster analysis (dl)
2
3
4
5
6
sample points
0
500
1000
1500
2000
48%
52%
48%
52%
45%
55%
55%
45%
55%
45%
50%
50%
36%
64%
55%
45%
50%
50%
36%
64%
1
2
3
4
5
6
clusters
sex
female
male

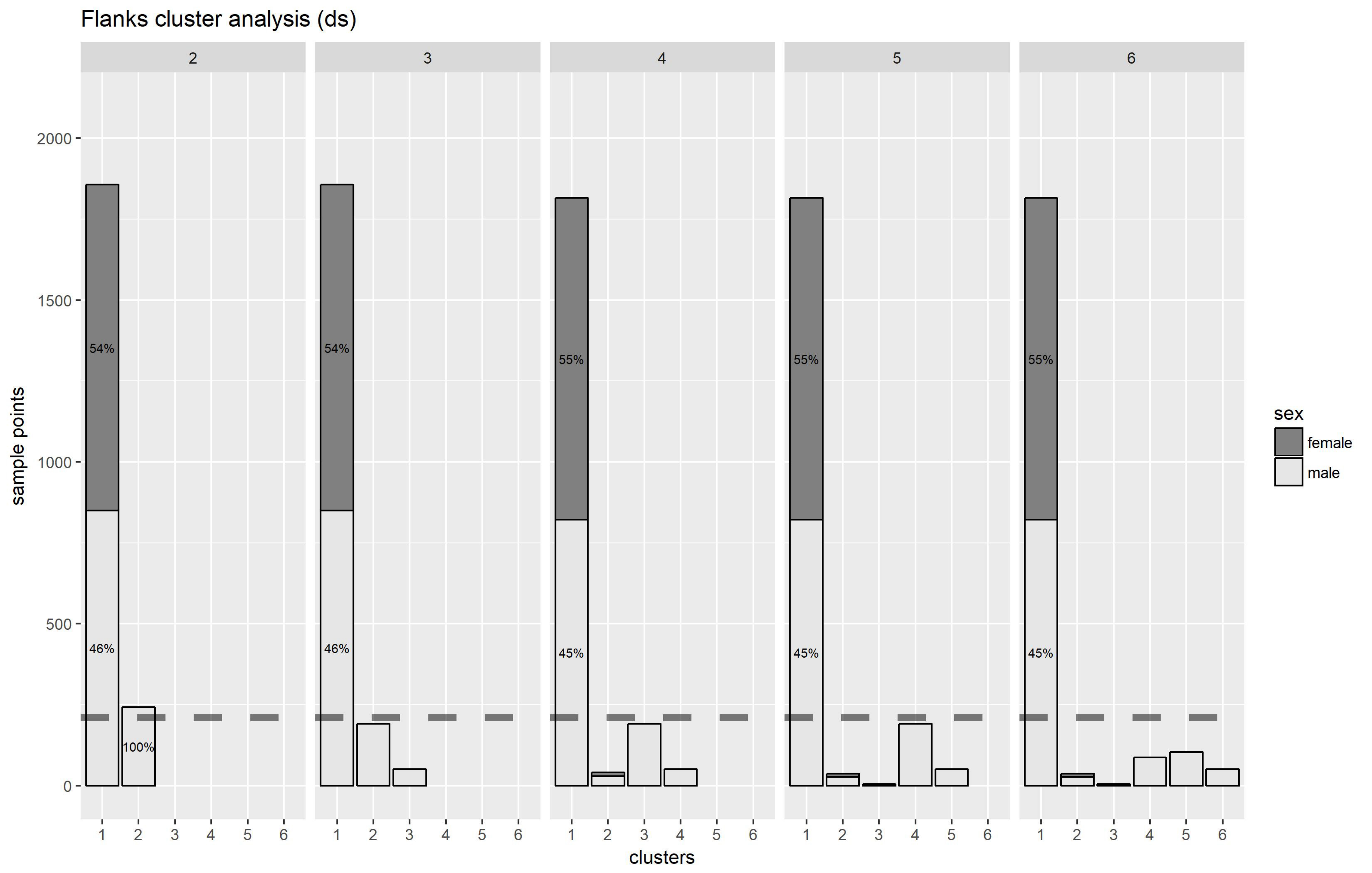
Flanks cluster analysis (ds)
2
3
4
5
6
sample points
0
500
1000
1500
2000
54%
46%
100%
54%
46%
55%
45%
55%
45%
55%
45%
clusters
1 2 3 4 5 6
sex
female
male

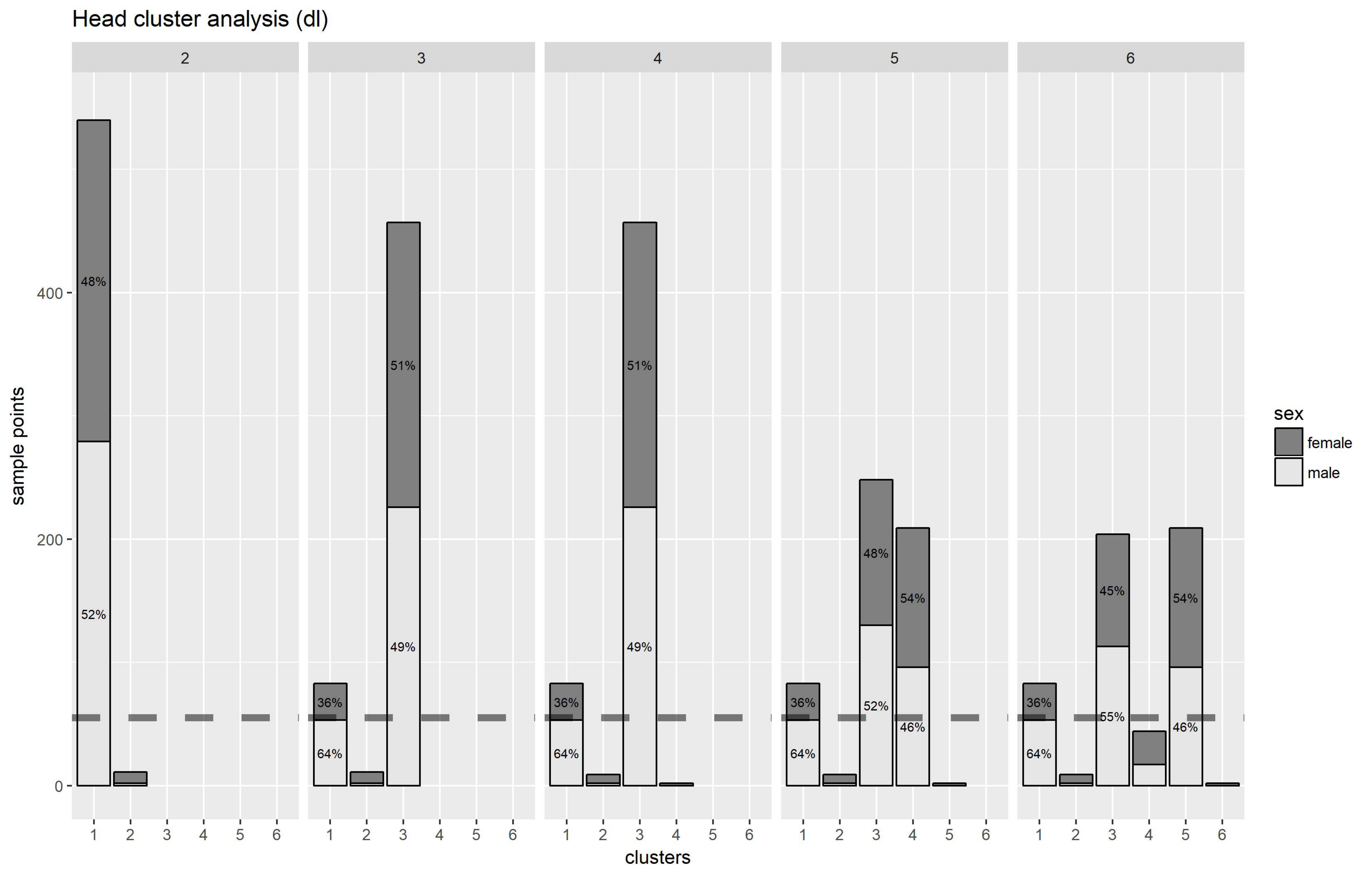
Head cluster analysis (dl)
2
3
4
5
6
48%
52%
51%
49%
36%
64%
51%
49%
36%
64%
48%
52%
54%
46%
36%
64%
45%
55%
54%
46%
36%
64%
400
200
0
sample points
1 2 3 4 5 6
clusters
sex
female
male

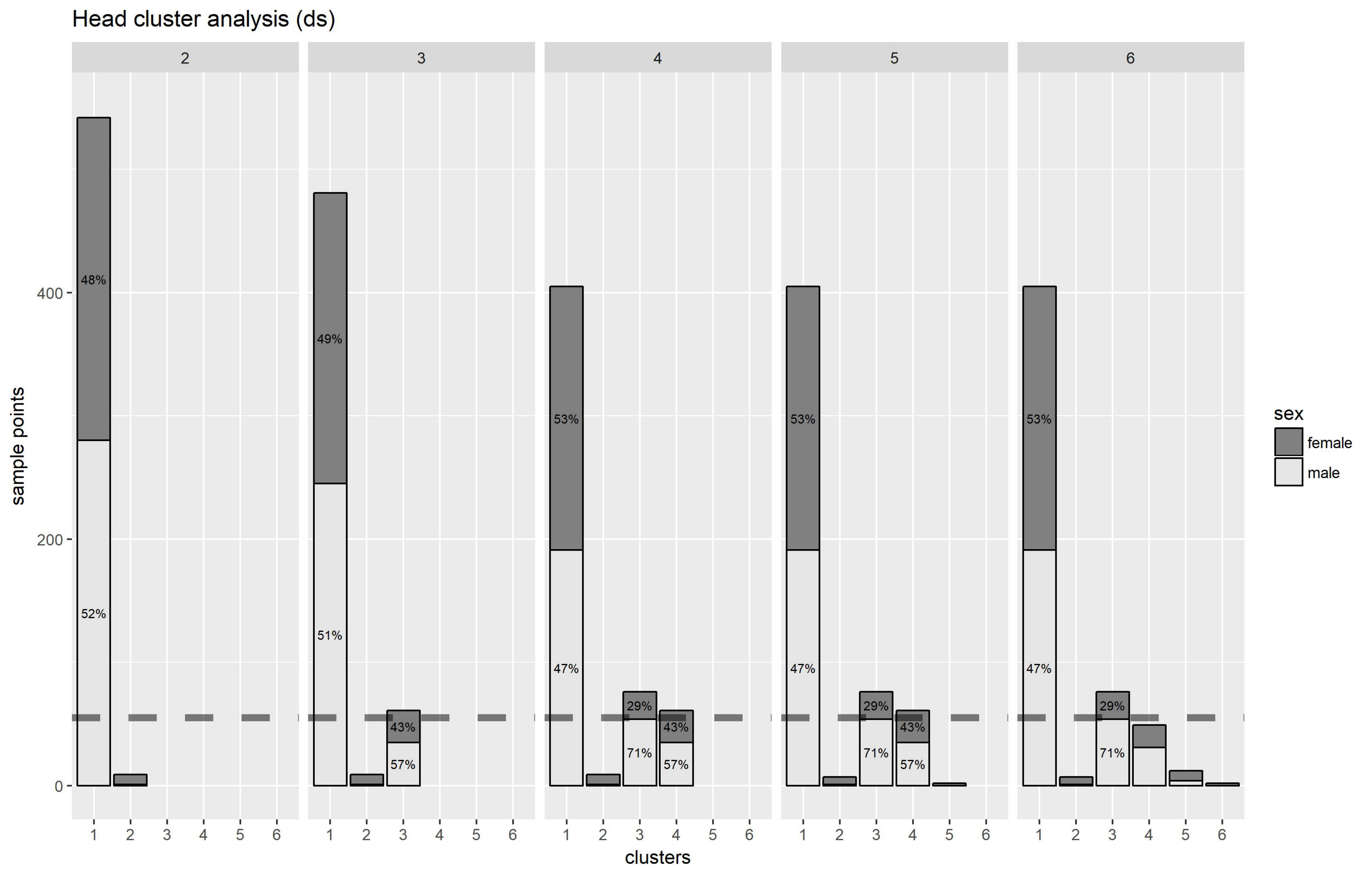

Head cluster analysis (ds)
2
3
4
5
6
sample points
0
200
400
48%
52%
49%
51%
43%
57%
53%
47%
29%
71%
43%
57%
53%
47%
29%
71%
43%
57%
53%
47%
29%
71%
clusters
1
2
3
4
5
6
sex
female
male

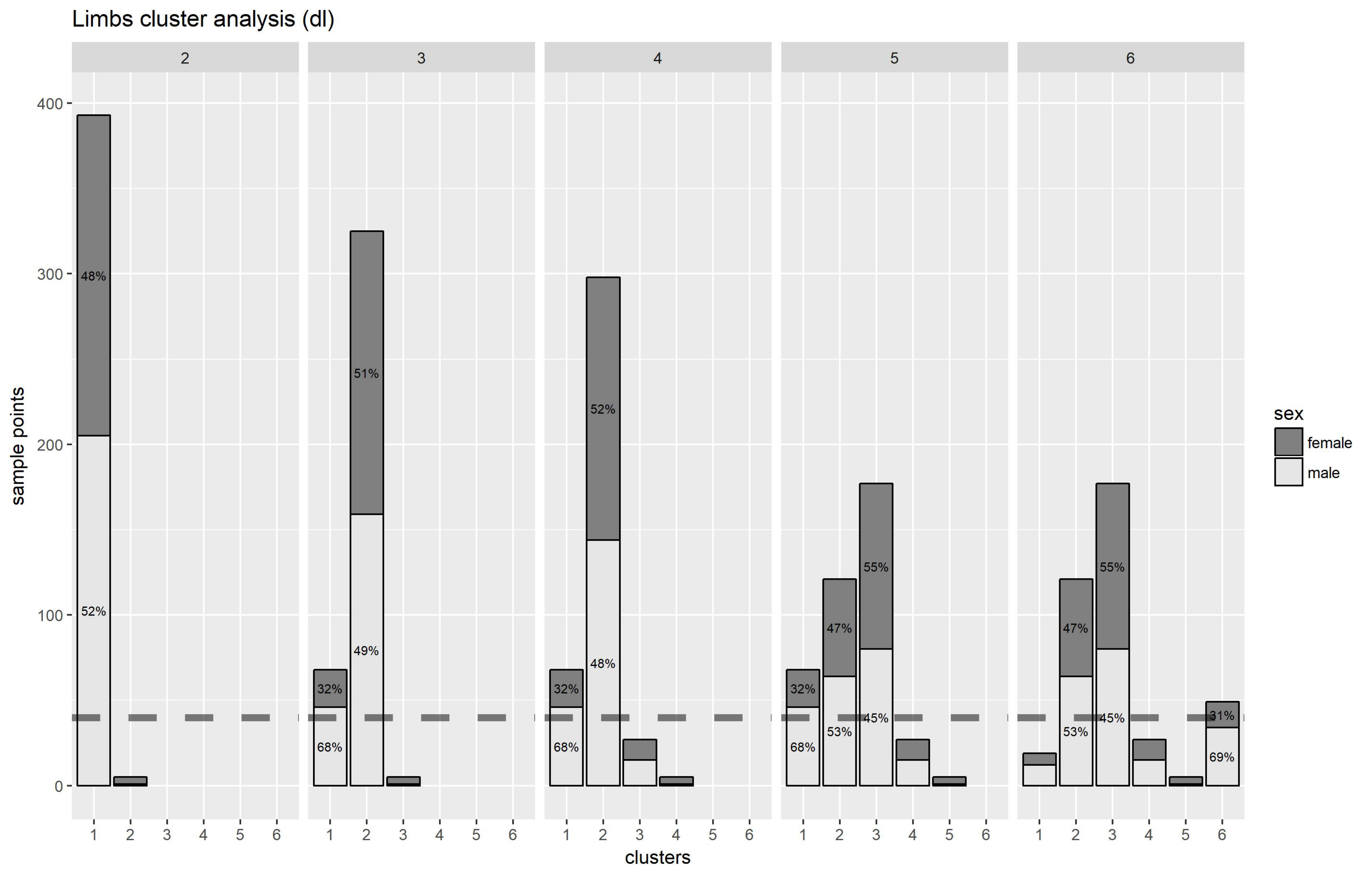

Limbs cluster analysis (dl)
2
3
4
5
6
sample points
clusters
sex
female
male
48%
52%
51%
49%
32%
68%
52%
48%
47%
55%
53%
45%
31%
69%

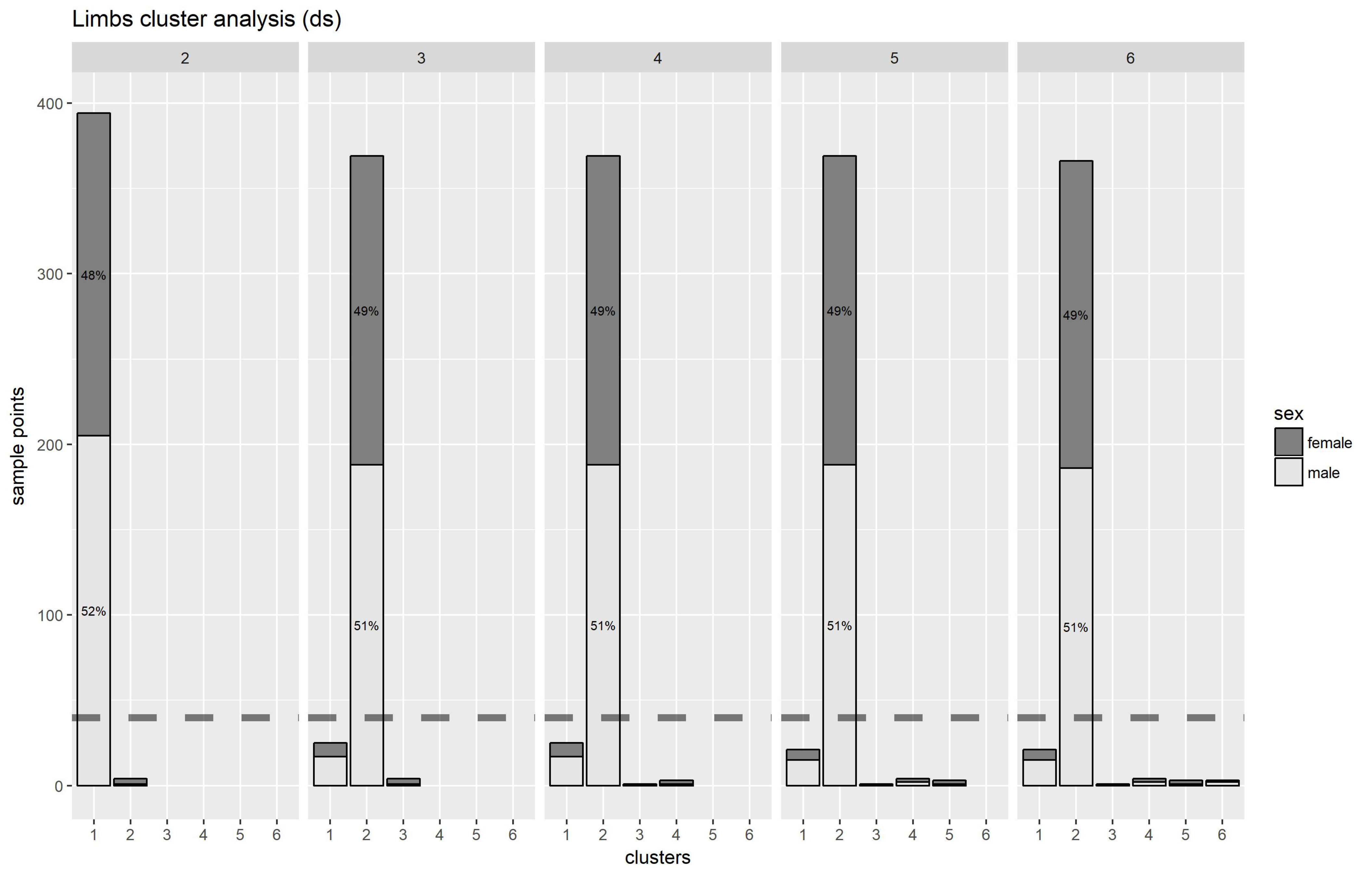

Limbs cluster analysis (ds)
2
3
4
5
6
400
300
200
100
0
sample points
48%
52%
49%
51%
49%
51%
49%
51%
49%
51%
1
2
3
4
5
6
clusters
sex
female
male

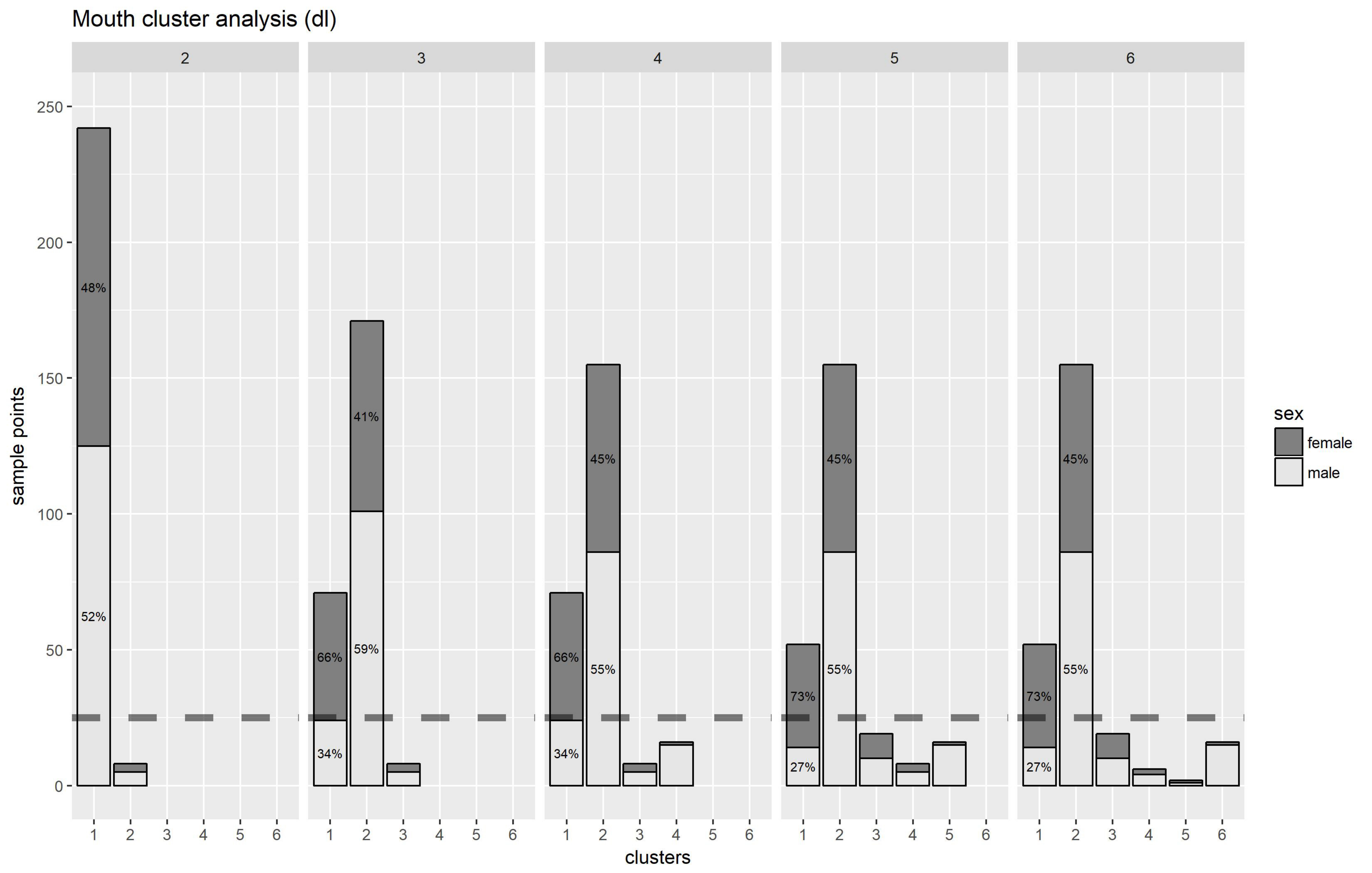

Mouth cluster analysis (dl)
2
3
4
5
6
250
200
150
100
50
0
sample points
48%
52%
41%
59%
66%
34%
45%
55%
66%
34%
45%
55%
73%
27%
45%
55%
73%
27%
1 2 3 4 5 6
clusters
sex
female
male

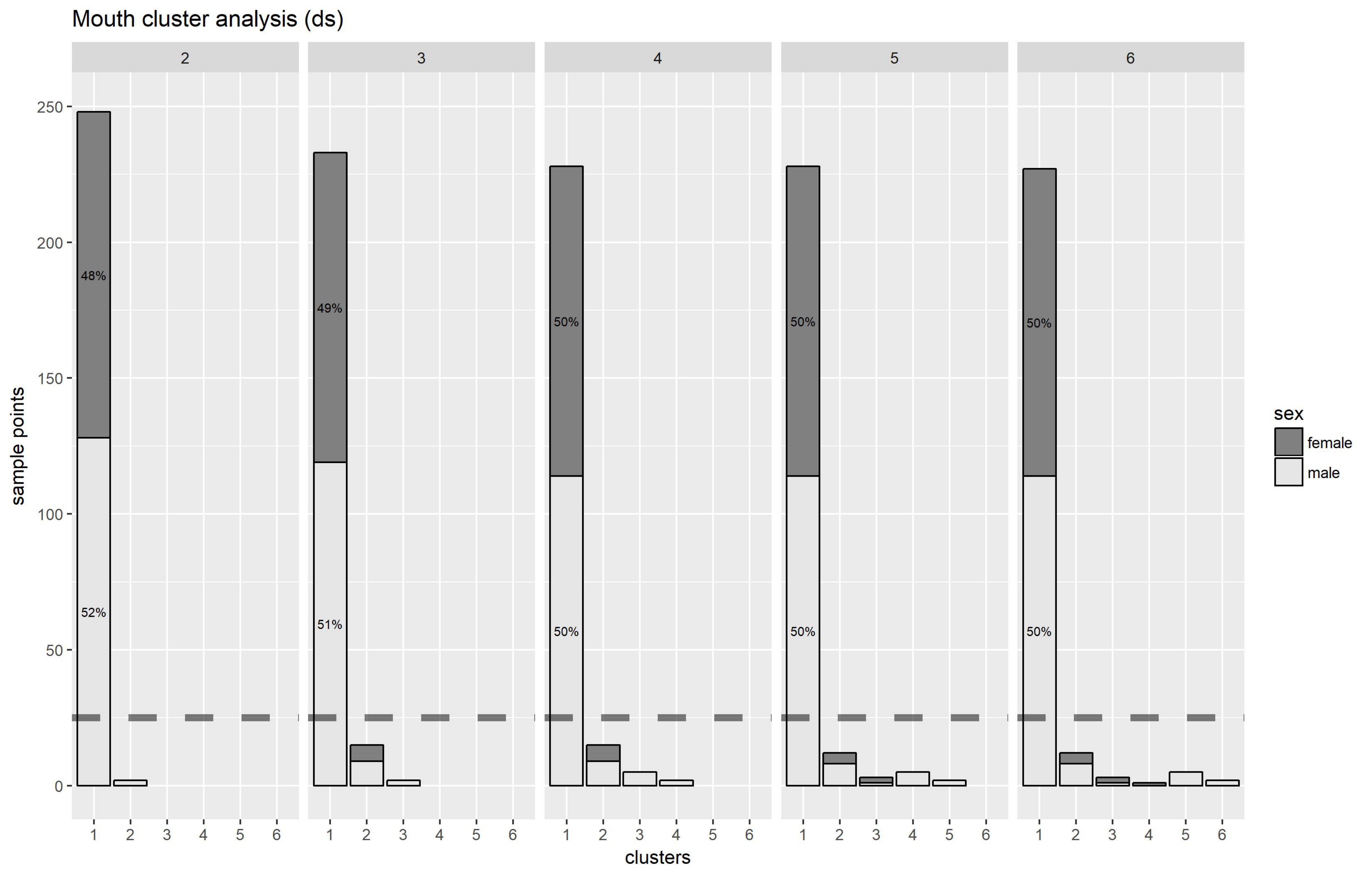

Mouth cluster analysis (ds)
2
3
4
5
6
sample points
clusters
sex
female
male
48%
52%
49%
51%
50%
50%
50%
50%
50%
50%

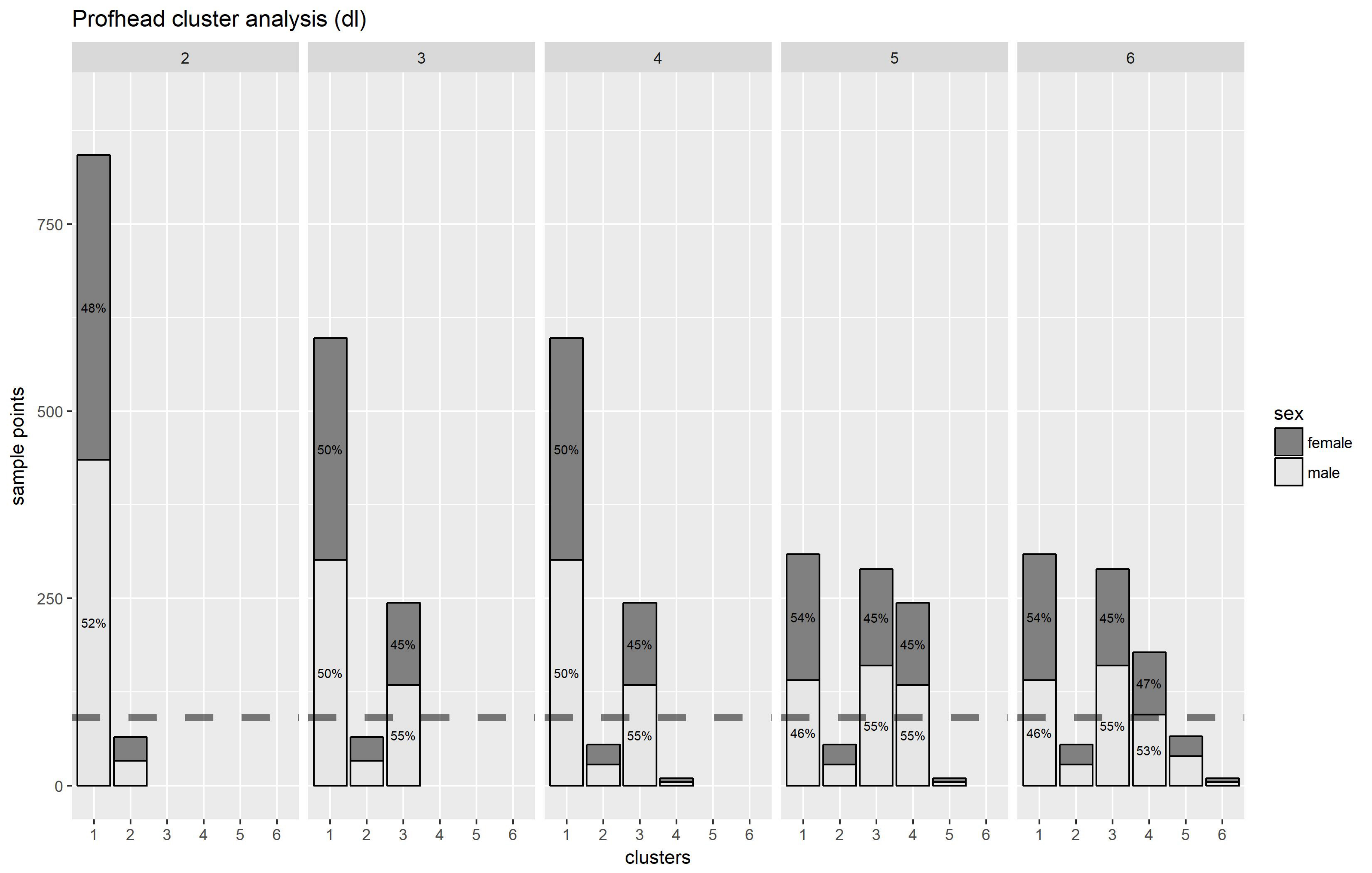

Profhead cluster analysis (dl)
2
3
4
5
6
sample points
750
500
250
0
clusters
1
2
3
4
5
6
48%
52%
50%
50%
45%
55%
50%
50%
45%
55%
54%
46%
45%
55%
45%
55%
54%
46%
45%
55%
47%
53%
sex
female
male

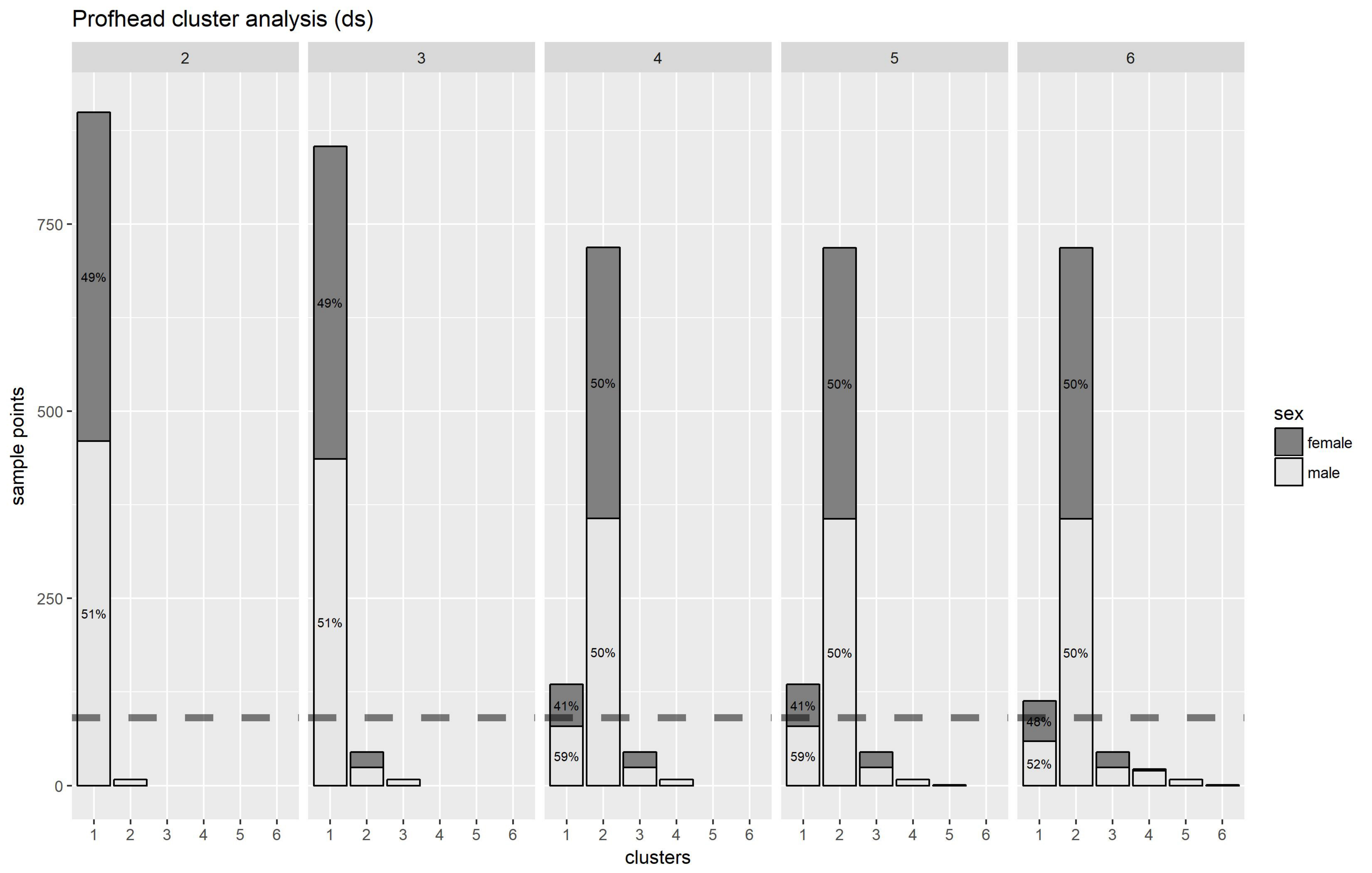
Profhead cluster analysis (ds)
2
3
4
5
6
sample points
0
250
500
750
clusters
1
2
3
4
5
6
49%
51%
41%
59%
50%
48%
52%
sex
female
male

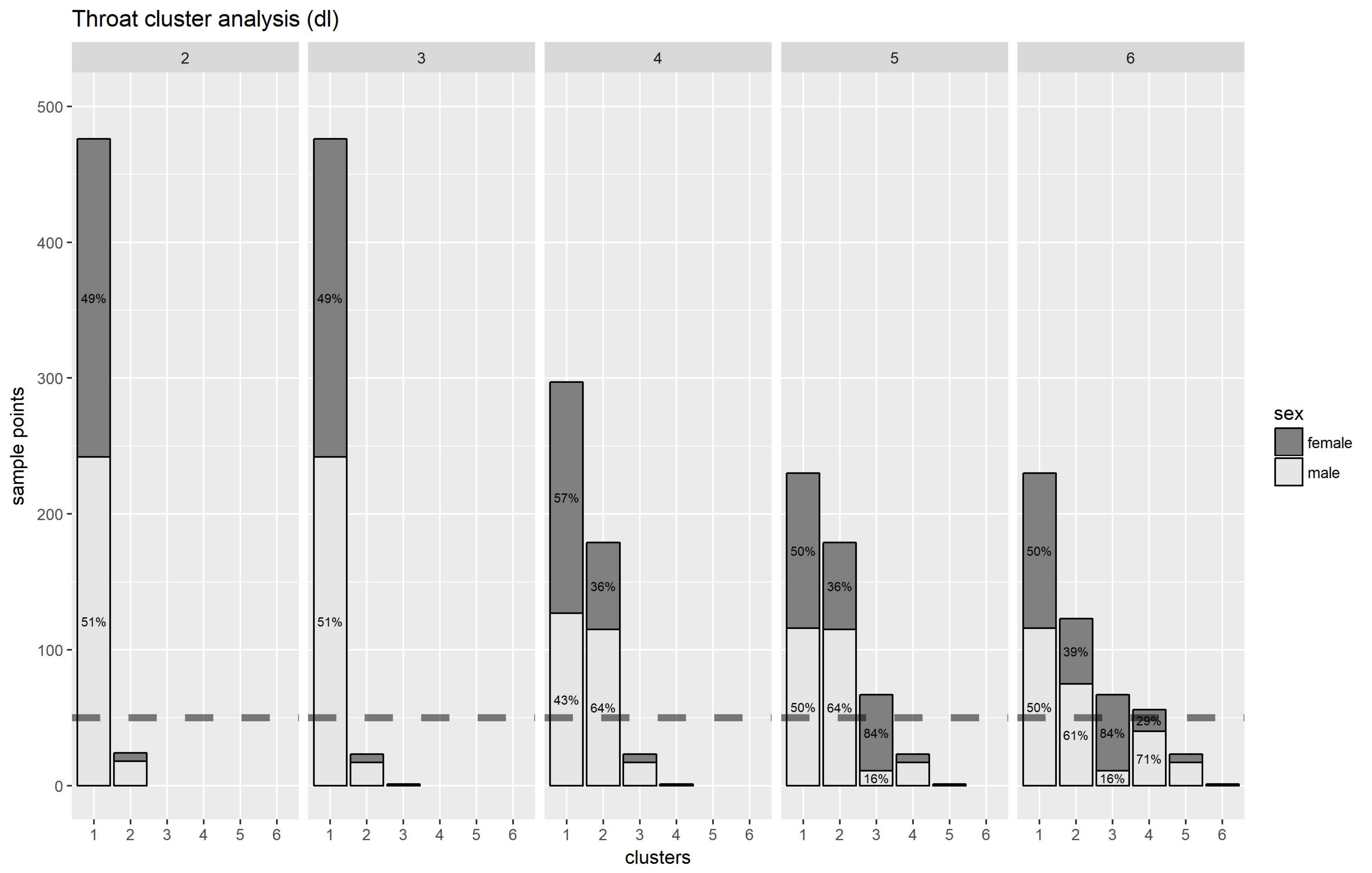
Throat cluster analysis (dl)
2
3
4
5
6
sample points
clusters
sex
female
male
0
100
200
300
400
500
1
2
3
4
5
6
49%
51%
57%
43%
36%
64%
50%
84%
16%
39%
61%
29%
71%

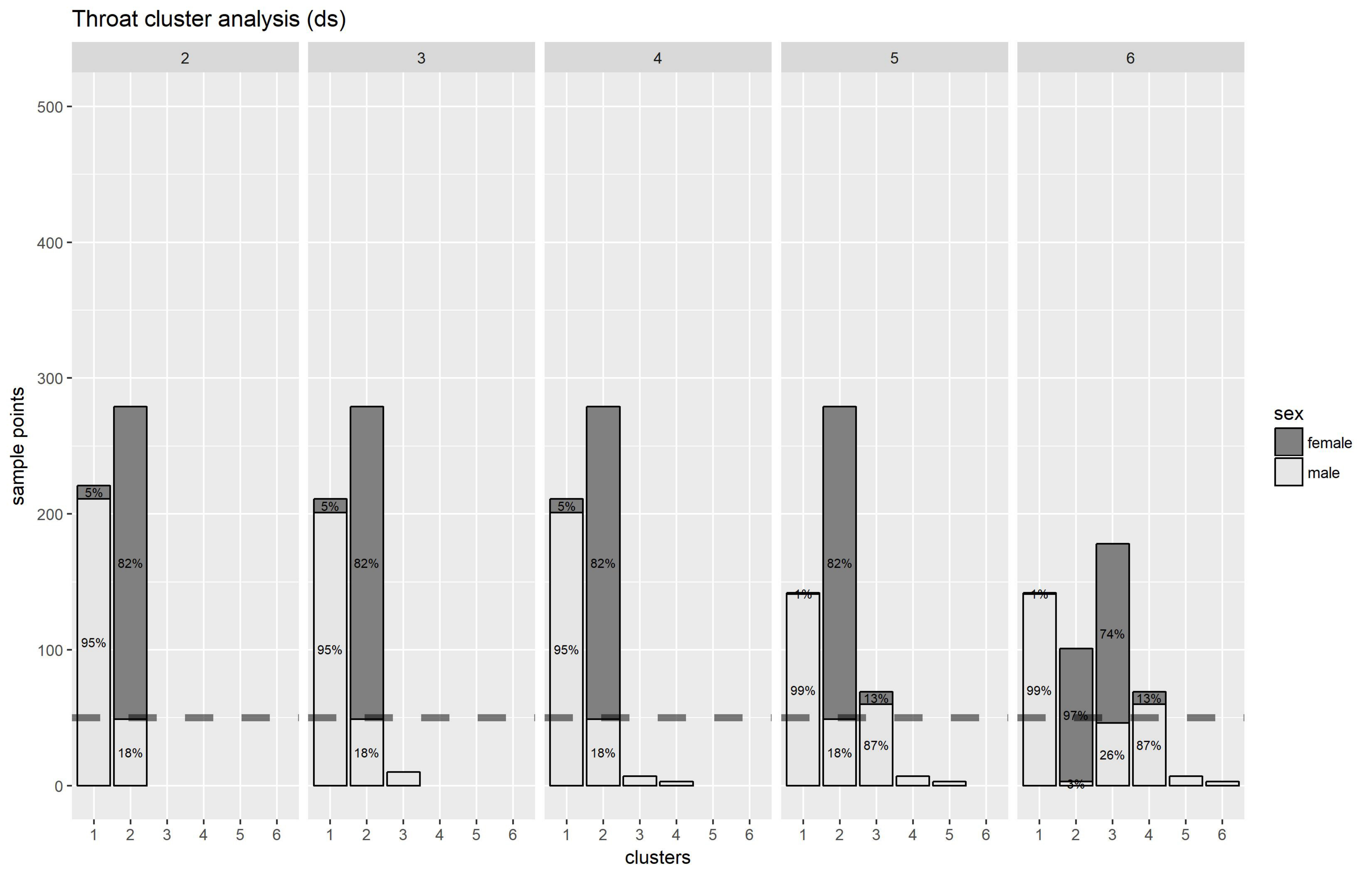

Throat cluster analysis (ds)
2
3
4
5
6
sample points
clusters
sex
female
male
5%
95%
82%
18%
99%
13%
87%
97%
74%
26%
3%

Supplementary figure S1: Cluster analysis plots showing clusters conformation for the 5 trees produced for each body region and type of contrast (dS and dL).

Bars height show the amount of spectra composing the cluster, the light grey section of bars indicate female spectra and dark grey male spectra; percentages within sections indicate the relative amount of spectra belonging to a sex (To be sex-specific, a cluster should be composed for the 90% of spectra of one sex). The dashed line in each graph corresponds to the 10% of the total amount of spectra for that region which was the second requisite for dichromatism. Plots were created using the package "ggplot" (Wickham et al. 2008).

Reference

Wickham H, Chang W et al. (2008) ggplot2: An implementation of the Grammar of Graphics. R package version 07, URL: http://CRAN R-project org/package= ggplot2

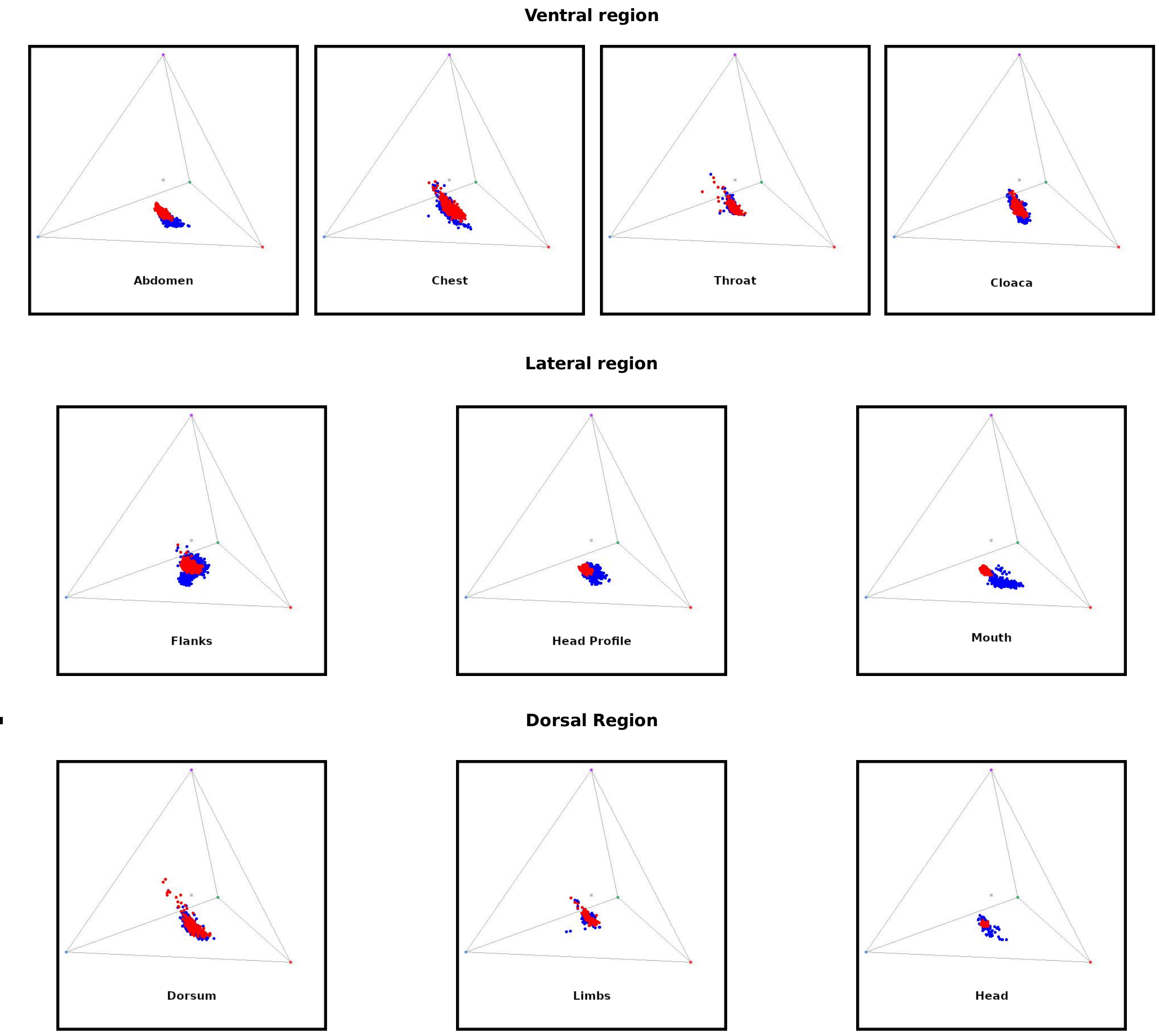
Ventral region
Abdomen
Chest
Throat
Cloaca
Lateral region
Flanks
Head Profile
Mouth
Dorsal Region
Dorsum
Limbs
Head

Supplementary figure S2: Plots of the tetrahedrical color space for each body region.

Coordinates within the tetrahedron correspond to the relative stimulation of the ultraviolet cone (violet vertex), short wavelength cone (blue vertex), medium wavelength cone (green vertex) and long wavelength cone (red vertex). Female coordinates are painted in red and males in blue. Plots were produced using the package “pavo” (Maia et al. 2013).

Reference:

Maia R, Eliason CM, Bitton P-P, et al (2013) pavo: an R package for the analysis, visualization and organization of spectral data. Methods in Ecology and Evolution 4:906–913

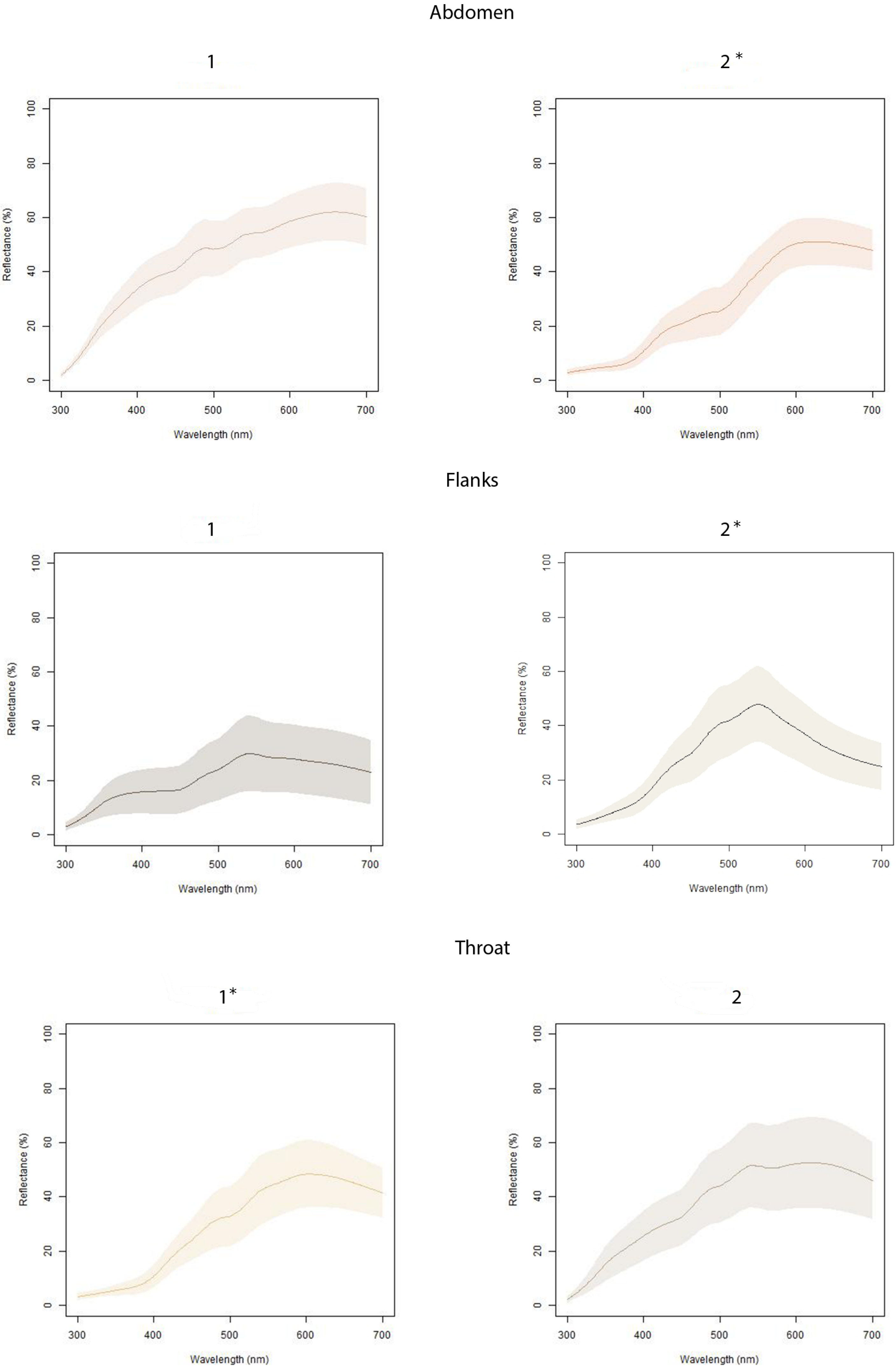

Abdomen
1
2*
Flanks
1
2*
Throat
1*
2
Reflectance (%)
Wavelength (nm)

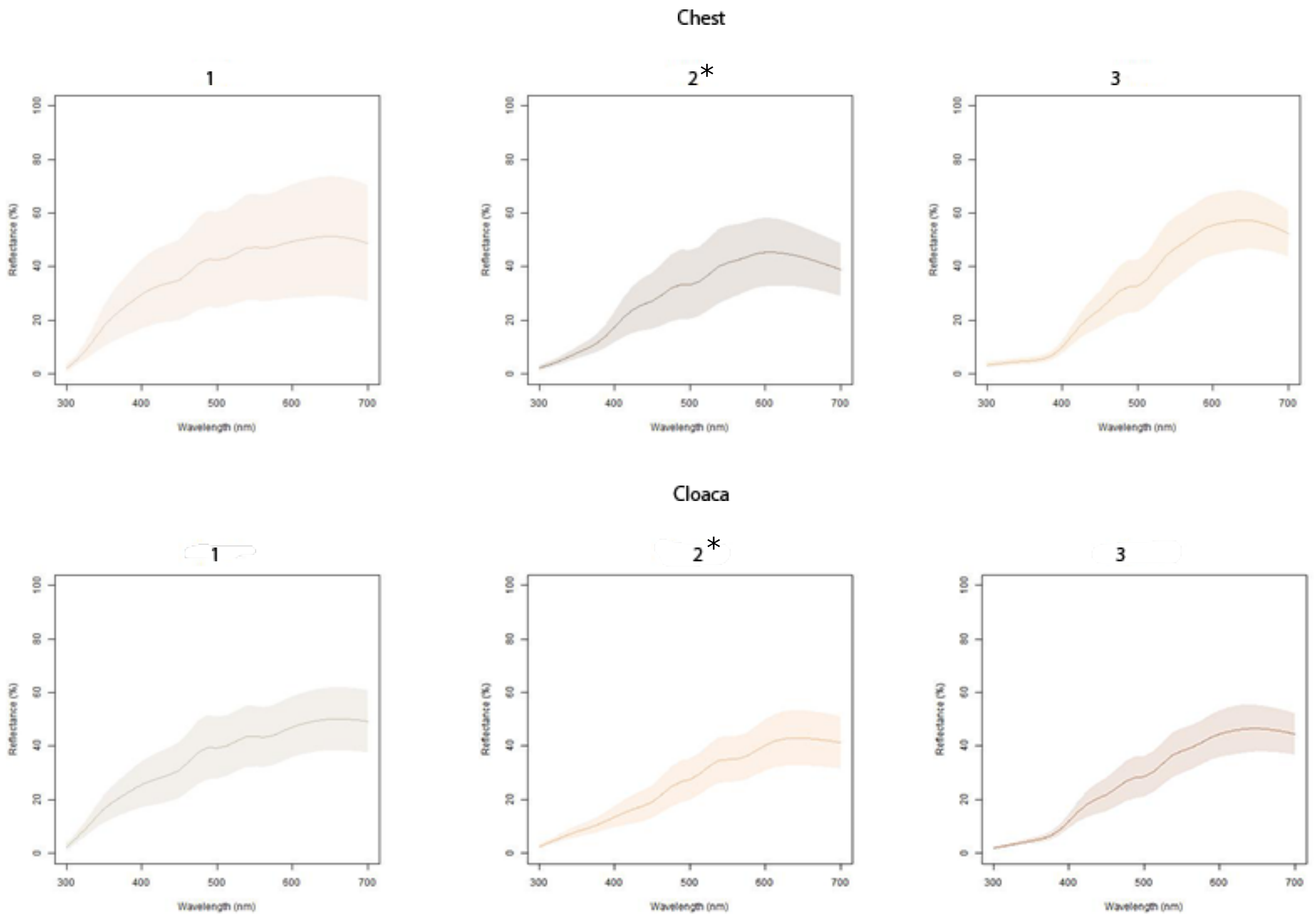
Chest
1
2*
3
Reflectance (%)
Wavelength (nm)
Cloaca
1
2*
3
Reflectance (%)
Wavelength (nm)

Supplementary figure S3: Agglomerative plots for the body regions that showed dichromatism according to the cluster analysis of Table 1.

Significant male-specific clusters of the final tree where marked with a *; thick lines indicate the mean spectra composing the regions while the softened areas indicate the standard deviation for each wavelength. Plots were produced using the package “pavo” (Maia et al. 2013) .

Reference:

Maia R, Eliason CM, Bitton P-P, et al (2013) pavo: an R package for the analysis, visualization and organization of spectral data. Methods in Ecology and Evolution 4:906–913
